# The Immuno-Regulatory Impact of Orally-Administered *Hypericum* *perforatum* Extract on Balb/C Mice Inoculated with H1n1 Influenza A Virus

**DOI:** 10.1371/journal.pone.0076491

**Published:** 2013-09-30

**Authors:** Nan Huang, Navrozedeep Singh, Kyoungjin Yoon, Christina M. Loiacono, Marian L. Kohut, Diane F. Birt

**Affiliations:** 1 The Center for Research on Botanical Dietary Supplements, Iowa State University, Ames, Iowa, United States of America; 2 Interdepartmental Graduate Program in Nutritional Sciences, Iowa State University, Ames, Iowa, United States of America; 3 Department of Food Science and Human Nutrition, Iowa State University, Ames, Iowa, United States of America; 4 Department of Kinesiology, Iowa State University, Ames, Iowa, United States of America; 5 Department of Veterinary Diagnostic and Production Animal Medicine, College of Veterinary Medicine, Iowa State University, Ames, Iowa, United States of America; 6 Pathobiology Laboratory- Pathology Section, National Veterinary Services Laboratories, US Department of Agriculture, Animal and Plant Health Inspection Service, Ames, Iowa, United States of America; The Ohio State University, United States of America

## Abstract

*Hypericum* *perforatum* ( *H* *. perforatum* ) ethanol extract has been found to inhibit lipopolysaccharide-induced production of inflammatory mediators and cytokines in cultured macrophages. Therefore, it may be able to protect the host from excessive inflammation during viral infection. In the current study, the immune-regulatory effect of *H* *. perforatum* extract was evaluated in A549 lung epithelial cells and BALB/c mice exposed to Influenza A/PR/8/34 H1N1 virus. In A549 cells, the extract (30 µg/mL) significantly inhibited influenza virus induced monocyte chemotactic protein (MCP)-1 and interferon-γ induced protein 10 kD (IP-10), but dramatically increased interleukin-6 (IL-6). In mice inoculated intranasally with 10^7.9^ EID_50_ of Influenza A/PR/8/34 H1N1 (high dose), daily oral treatment of *H* *. perforatum* extract at a rate of 110 mg/kg of body weight increased lung viral titer, bronchoalveolar lavage (BAL) pro-inflammatory cytokine and chemokine levels, and the infiltration of pro-inflammatory cells in the lung 5 days post-inoculation, as compared to ethanol vehicle treated mice. Transcription of suppressor of cytokine signaling 3 (SOCS3) was increased by *H* *. perforatum* extract both in A549 cells and BALB/c mice, which could have interrupted anti-viral immune response and thus led to the inefficient viral clearance and increased lung inflammation. *H* *. perforatum* treatment resulted in minor reduction in viral titer without affecting body weight when mice were inoculated with a lower dose (~10^5.0^ EID_50_) and *H* *. perforatum* was applied in the later phase of infection. Mice challenged intranasally with high dose of influenza virus (10^7.9^ EID_50_) suffered from a higher mortality rate when dosed with *H* *. perforatum* extract. In conclusion, the current study showed that SOCS3 elevation by *H* *. perforatum* may cause impaired immune defense against influenza virus infection and lead to higher mortality.

## Introduction

Influenza virus has been a major public health burden for centuries, affecting 10-20% of the general population and causing approximately 36,000 deaths annually in the United States [1,2]. Despite enormous vaccination efforts, influenza seasons persist and a much feared potential outbreak of pandemic influenza like the one in 1918 could result in a mortality of over 80 million according to statistical predictions using regression analysis [1]. Upon contracting influenza virus, the host immune system is activated to contain and resolve the infection. Respiratory epithelial cells secrete a wide variety of pro-inflammatory cytokines and chemokines that attract and activate innate immune cells, which subsequently initiate adaptive immune mechanisms to clear viral particles [3-5]. Although cytokines can inhibit viral replication and are critical for the immune response, pro-inflammatory cytokines and inflammatory immune cells also contribute to pneumonia and tissue damage [6]. Certain strains of influenza virus, such as H5N1, are more likely to induce excessive cytokine release and immune cell exudation [4]. This so-called ‘cytokine storm’ scenario, features elevated levels of cytokines and chemokines such as tumor necrosis factor (TNF)-α, interleukin (IL)-6, monocyte chemotactic protein (MCP)-1, and interferon (IFN)-γ, as well as the exudation of monocytes, macrophages, and neutrophils. ‘Cytokine storm’ causes tissue damage, impairs normal mucosal membrane and may induce airway blockage, making it a risk factor for the higher mortality associated with these virulent strains [7,8]. Therefore, alleviating inflammation during influenza virus infection could potentially be beneficial.

*Hypericum* *perforatum* ( *H* *. perforatum* ) is a perennial medicinal plant primarily used by patients with depression disorders [9]. Its ethanol extracts have also been shown to have anti-viral and anti-inflammatory activities [10,11]. Our previous research demonstrated that *H* *. perforatum* ethanol extract inhibited LPS-induced production of inflammatory mediators including prostaglandin E2 (PGE2) and nitric oxide (NO) in activated macrophages. One objective of this study was to determine whether *H* *. perforatum* extract can inhibit influenza virus-stimulated production of pro-inflammatory cytokine and/or levels of cytokine production.

Suppressor of cytokine signaling 3 (SOCS3) is an intracellular negative regulator of the Janus kinase-signal transducer and activator of transcription (JAK-STAT) signaling pathway [12]. Its function has been described as inhibitory against inflammation because it inhibits the JAK-STAT, mitogen-activated protein kinase (MAPK) and toll-like receptor (TLR) pathways directly or/and indirectly [13-15]. Some evidence suggests a role of SOCS3 in IL-6 signaling [16]. IL-6, which usually promotes pro-inflammatory TNF-α and IL-12 production in LPS-induced activated macrophages, was found to inhibit these cytokines when the expression of SOCS3 gene was absent. Previously, we found that SOCS3 was elevated by treatment of macrophages with *H* *. perforatum* extract, and this elevation may partially account for the observed anti-inflammatory potential of four major active compounds in the extract [17]. However, recent studies suggested that H1N1 virus suppressed the innate immune response by increasing SOCS3 expression and the subsequent JAK-STAT signaling inhibition in BEAS-2B cells (transformed human bronchial epithelial cells) [18]. If this observation reflects what is seen *in vivo*, SOCS3 elevation may allow higher viral titer and less efficient viral clearance.

In the current study, we addressed the impact of *H* *. perforatum* ethanol extract on H1N1 influenza virus inoculated human alveolar epithelial cell line A549 and BALB/c mice, with particular focus on cytokine production, inflammatory damage, viral titer, and SOCS3 gene alteration.

## Materials and Methods

### Ethics Statement

All animal usage procedures in strict accordance with the recommendations in the Guide for the Care and Use of Laboratory Animals of the National Institutes of Health and were approved by the Iowa State University Institutional Animal Care and Use Committee (Protocol 10-06-6238-R).

### *H* *. perforatum* extract

Procurement and extraction of *H* *. perforatum* plant material were as previously described [19]. In brief, 6 g of dry *H* *. perforatum* (Accession PI325351) plant material, acquired from the North Central Regional Plant Introduction Station (NCRPIS) (Ames, IA) of the U.S. Department of Agriculture, Agricultural Research Service (USDA/ARS) was ground and extracted with 500 mL of 95% ethanol using Soxhlet extraction. The extract was dried and weighed before it was dissolved in pure DMSO or 50% ethanol (both from Sigma, St. Louis, MO). Known chemical constituents were quantified and the extract was stored at -20 °C in the dark.

### A549 epithelial cells

A549 human bronchial alveolar epithelial cells were acquired from American Type Culture Collection (ATCC, Manassas, VA). Cells were maintained in F12K media supplemented with 10% fetal bovine serum, 100 IU/mL penicillin/ 100 µg/mL streptomycin, 0.25 µg/mL amphotericin B, and 50 µg/mL gentamicin (all purchased from Life Technologies, Carlsbad, CA).

### H1N1 influenza virus

Influenza A/PR/8/34 H1N1 virus was grown in the amniotic-allantoic sac of 10-11 day old embryonated eggs. The haemagglutination unit (HAU) of the virus stock was 4096 HAU/0.05 ml (or 10^10.45^ EID_50_/ml).

### A549 cell viral challenge and treatments

Influenza A/PR/8/34 H1N1 virus was diluted to 10^7.8^ EID_50_/mL, in serum free media, and applied to A549 cells at 1 × 10^6^ cell/well in 24-well plates, or 4 × 10^6^ cell/well in 6-well plates. The volume of virus-containing media was 50 µL/well for 24-well plates and 200 µL/well for 6-well plates. After 1 hr of incubation, 450 µL (24-well plates) or 1.8 mL (6-well plates) of media containing DMSO vehicle control or 30 µg/mL H*. perforatum* extract in DMSO (DMSO accounted for 0.1% of the total final volume) were added to each well and maintained until the end of the 3-hr or 24-hr experiments, when the supernatant or cells were collected for further analysis.

### A549 cell viability determination

After the supernatant was collected CellTiter 96® AQueous One Solution was added to the cells with fresh media according to the manufacturer’s instruction (Promega, Madison, WI). After 3 hours and 15 minutes of incubation, optical absorbance (490 nm) was quantified to determine cell viability.

### ELISA for A549 cell culture supernatant

Collected supernatant samples were subjected to enzyme-linked immunosorbent assay (ELISA) to determine IL-6, TNF-α, MCP-1, and IP-10 levels. ELISA kits for these cytokines were used following manufacturer’s instruction, after diluting samples to concentrations within the range of standard curves (BD Biosciences, Franklin Lakes, NJ).

### Extraction of A549 cell RNA and gene transcription measurements

A549 cells were harvested for RNA extraction using the Trizol method [19]. RNA was subsequently purified with RNeasy kit (Qiagen, Valencia, CA) and reverse-transcribed into cDNA using an iScript cDNA synthesis kit, followed by gene transcription quantification using an iCycler coupled with a MyiQ optical module (all from BioRad, Hercules, CA). Primers used for this quantitative real-time polymerase chain reaction (qRT-PCR) were obtained from Integrated DNA Technologies, Inc. (Coralville, IA). The SOCS3 primer sets were 5’-ATT CGC CTT AAA TGC TCC CTG TCC-3’ (forward) and 5’- TGG CCA ATA CTT ACT GGG CTG ACA-3’ (reverse); 5’-TCG ACA GTC AGC CGC ATC TTC TTT -3’ (forward) and 5’- ACC AAA TCC GTT GAC TCC GAC CTT-3’ (reverse) for the housekeeping gene glyceraldehydes 3-phosphate dehydrogenase (GAPDH).

### Extraction of A549 cell protein and Western blot

A549 cells were collected 24 hour after infection with virus, and subjected to protein extraction as previously described [19]. Western blotting was conducted using antibodies against SOCS3 (sc-51699) and GAPDH (sc-137179, all from Santa Cruz Biotechnology, Santa Cruz, CA) [17].

### Mice and gavage treatments

Male BALB/c mice aged between 6-10 weeks were used in this study (Charles River Laboratory, MA). Upon arrival, mice were allowed to acclimate to the environment for 1+ week before being subjected to the experiments. Mice were assigned into different treatment groups randomly with body weight equalized among groups before treatments began. Mice were kept in individual cages at 25 °C and 40% humidity under 12-hr light/dark cycles, with free access to normal rodent chow diet (Harlan Teklad 2014) and tap water. Daily treatments of 5% ethanol vehicle control, or *H* *. perforatum* extracts at different concentrations in 150 µL of 5% ethanol were administrated orally using 18 gauge animal feeding needles (Cadence Science, Staunton, VA). A total of four studies were conducted. In the two short term studies, including a 6-day (10^5.0^ EID_50_ or 10^5.1^ EID_50_/mouse; low viral titer) and a 5-day (10^7.9^ EID_50_/mouse; high viral titer) inoculation experiments, gavage was conducted at the same time daily from one day before inoculation (day -1) to the day before animal sacrifice (day 5 and day 4). Gavage was administrated from day 5 post-inoculation (PI) to the day before the necropsy in the 10-day low viral titer infection study (i.e., day 5 to day 9 PI). For the high viral titer survival study, gavage was administered daily from day -1 PI until the sacrifice of animal.

### Toxicity of *H* *. perforatum* extract in BALB/c mice

Toxicity of the extract was tested by gavaging mice with 5% ethanol, 60 mg/kg, 110 mg/kg, or 220 mg/kg body weight of *H* *. perforatum* ethanol extract daily for 3 wks. Body weight and food and water consumption were monitored daily over the experiments, with heart, liver, spleen, kidney, and stomach weight recorded at the end of the study during necropsy.

### Mouse influenza viral infection and health monitoring

Mice were inoculated with A/PR/8/34 H1N1 influenza virus on day 0 of each study. The procedure involved anesthesia with isoflurane (VAD Anesthetic Machine from Vetamac, Rossville, IN) followed by inoculation of 30 µL virus in saline intranasally. Viral doses were 10^5.0^ EID_50_ or 10^5.1^ EID_50_ for the low viral titer studies, and 10^7.9^ EID_50_ for the high titer studies. Daily records of mouse body weight, as well as food and water disappearance (as crude measurement of consumption) were collected, except on days towards the end of some studies, when body weight loss exceeded 15%. In these cases, with the mice being too weak to reach the food and water on the cage top, food and water were supplied in petri dishes to allow easier access.

### Mouse illness score assessment

Mice were evaluated by an observer blinded to the treatments for their sickness condition after inoculation using a scoring system adopted from Murphy et al., which is based on ruffled hair/ hunch back, eye and nose redness, and unresponsiveness [20]. Each of the three signs was given a score of 0 to 2 (for the most severe) according to severity. The average of scores in three categories was used as the overall illness score for each individual mouse.

### Necropsy and bronchoalveolar lavage collection

Mice were euthanized at the end of the studies using carbon dioxide. Blood was collected through heart puncture. Bronchoalveolar lavage (BAL) fluid was collected and processed for subsequent flow-cytometry, cytokine, chemokine, and NO assays as described by Sim et al. [21].

### BAL cytokine and nitric oxide measurement

BAL supernatant, collected after centrifugation, was analyzed for cytokine and chemokine levels on a Luminex platform (Bio-Rad) with a MILLIPLEX map mouse 32-cytokine/chemokine multiplex kit (Millipore, Billerica, MA). The panel included eotaxin, granulocyte colony-stimulating factor (G-CSF), granulocyte macrophage colony-stimulating factor (GM-CSF), IFN-γ, IL-1α, IL-1β, IL-2, IL-2, IL-4, IL-5, IL-6, IL-7, IL-9, IL-10, IL-12 (p40), IL-12 (p70), IL-13, IL-15, IL-17, IP-10, keratinocyte-derived cytokine (KC), leukemia inhibitory factor (LIF), LPS-stimulated CXC chemokine (LIX), M-CSF, MCP-1, monokine induced by IFN-γ (MIG), macrophage inflammatory protein-1α (MIP-1α), MIP-1β, MIP-2, regulated upon activation, normal T-cell expressed, and secreted cytokine (RANTES), TNF-α, and vascular endothelial growth factor (VEGF). NO was measured using Greiss reagent (Promega, Madison, WI) as previously described [22].

### BAL cell population characterization

BAL cells were stained for conjugated antibodies against different surface proteins. Antibodies used included FITC-anti-mouse CD8b, Alexa Fluor® 647-anti-mouse CD11b, and PE-anti-mouse Gr1 (eBioscience, San Diego, CA). Labeled samples, along with isotype controls, were subject to BD FACS Canto flow cytometer analysis (BD Biosciences). Cell populations were characterized according to forward scatter, side scatter, and fluorescent signals using FlowJo software (Tree Star, Ashland, OR). Specifically, CD11b^+^, GR1^+^, and high side scatter (SSC) BAL cells were considered as neutrophils in the 6-day low viral titer study, while cytotoxic T cells were CD8β^+^, and mononuclear phagocytes being GR1^intermediate^ with low SSC. In the 5-day high viral titer study, neutrophils were CD11b^+^ and GR1^+^ with high SSC; cytotoxic T cells were CD8β^+^; inflammatory macrophages were CD11b^+^and GR1^-^ with high auto-fluorescence [23]; resident alveolar macrophages were CD11b^-^ and GR1^-^ with high auto-fluorescent; inflammatory monocytes were CD11b^+^ and GR1^intermediate^ with low SSC [24].

### Lung index, histopathology score, and viral titer quantification

Lungs were harvested from the mice and weighed in the 6-day low viral titer and 5-day high viral titer infection studies. Lung index, a crude measurement of lung inflammation during infection, was calculated using the formula: (lung weight)/(body weight) × 100 [25]. In the 10-day low viral titer infection study, 4 lobes of lung were fixed in buffered formalin (Fisher Scientific, CA), followed by paraffin embedding. Approximately 5 µm thick sections were stained with hematoxylin-eosin and inspected under a light microscope. Lung histophathological lesions, characterized by necrosis, degeneration, hyperplasia, and infiltration, were evaluated and scored blinded to treatments using standards previously used by Sim et al. [21]. Lung viral titer was determined using qRT-PCR using specific primers against conservative nucleoprotein gene as described in detail by Sim et al. [21].

### Lung RNA extraction and gene transcription quantification

One lobe of lung from each mouse was snap-frozen upon necropsy and ground in liquid nitrogen. The ground tissue was combined with Trizol reagent (Invitrogen) and homogenized. Trizol method and RNeasy (Qiagen) purification procedures were subsequently applied to obtain purified RNA from lung tissue. RNA samples were then reverse-transcribed into cDNA using an iScript cDNA synthesis kit (BioRad). cDNA samples were normalized to the same 100 ng/mL concentration before subjection to gene transcription quantification in iCycler coupled with a MyiQ optical module (BioRad). Primers used were 5’-ATT CAC CCA GGT GGC TAC AG-3’ (forward) and 5’-GCC AAT GTC TTC CCA GTG TT-3’ (reverse) for SOCS3, as well as 5’-CAA TGT GTC CGT CGT GGA T-3’ (forward) and 5’-AGC CCA AGA TGC CCT TCA G-3’ (reverse) for GADPH.

### Mouse survival after high dose influenza virus inoculation

Mice were monitored for their body weight during the survival study. When body weight loss exceeded 20% of initial level, the mouse was euthanized. Survival or mortality was assessed until day 10 PI.

### Statistical analysis

Measured A549 cell cytokine production and mRNA transcription levels from 3 replicates of culture plates in the same experiment were log-transformed and analyzed using ANOVA as a randomized complete block design with cell culture plates as fixed blocks. All treatments with or without viral infection were compared to the media + DMSO vehicle control. Animal study data that was repeatedly measured, such as body weight, food and water intake, and illness score, were analyzed using a mixed ANOVA model that included an unspecified structure for the day to day repeat correlation. For post-hoc tests, treatments were compared to each other on each day after *Tukey* adjustment. Lung index, cell population, lung lesion score, log-transformed cell viral titer and lung gene transcription levels were analyzed using ANOVA test, followed by multiple comparisons between individual treatment groups with *Tukey* adjustment (SAS 9.0, SAS Institute, Cary, NC). Survival data was analyzed by comparing Kaplan-Meier survival curve between the vehicle control and *H* *. perforatum* groups in SPSS (SPSS 17.0, SPSS Inc., Chicago, IL).

## Results

### Cytokine released by A549 lung bronchial epithelial cells

**Figure 1:**
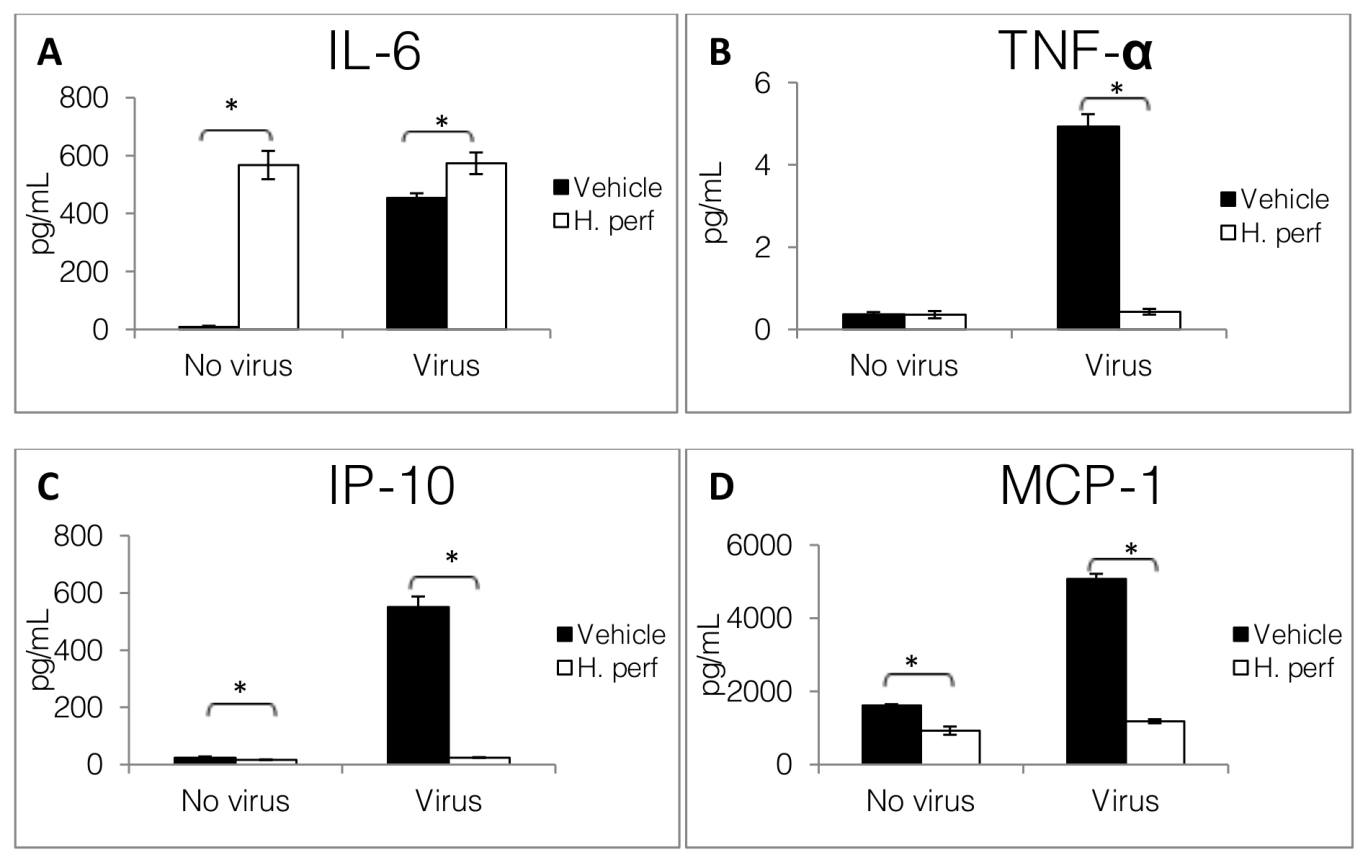
Cytokines released by A549 human bronchial epithelial cells. DMSO vehicle control or 30 µg/mL *H*. *perforatum* extract in DMSO were applied to cells, with or without H1N1 influenza virus inoculation. Cytokine levels for IL-6 (A), TNF-α (B), IP-10 (C), and MCP-1 (D) after 24 hrs of treatment are shown as Mean ± SEM (N=3). Significant difference between vehicle and *H*. *perforatum* extract treatments are noted with * (p<0.05).

Production of IL-6, IP-10, TNF-α, and MCP-1 by A549 cells were drastically stimulated 24 hrs after H1N1 virus inoculation as shown in Figure **1**. In comparison to DMSO vehicle control, *H* *. perforatum* treatment significantly increased IL-6 production (**panel A**) in cells without virus inoculation, resulting in a level even higher than that of inoculated vehicle treated cells. Virus-induced TNF-α (**panel B**), IP-10 (**panel C**), and MCP-1 (**panel D**) production were inhibited by *H* *. perforatum* extract treatment. The background levels of IP-10 and MCP-1 produced by A549 cells were also decreased when treated with *H* *. perforatum* extract. No apparent difference in cell viability was found associated with virus challenge or the treatment of extract at the 24 hr time point (Figure **S1**).

### SOCS3 expression in A549 cells

**Figure 2:**
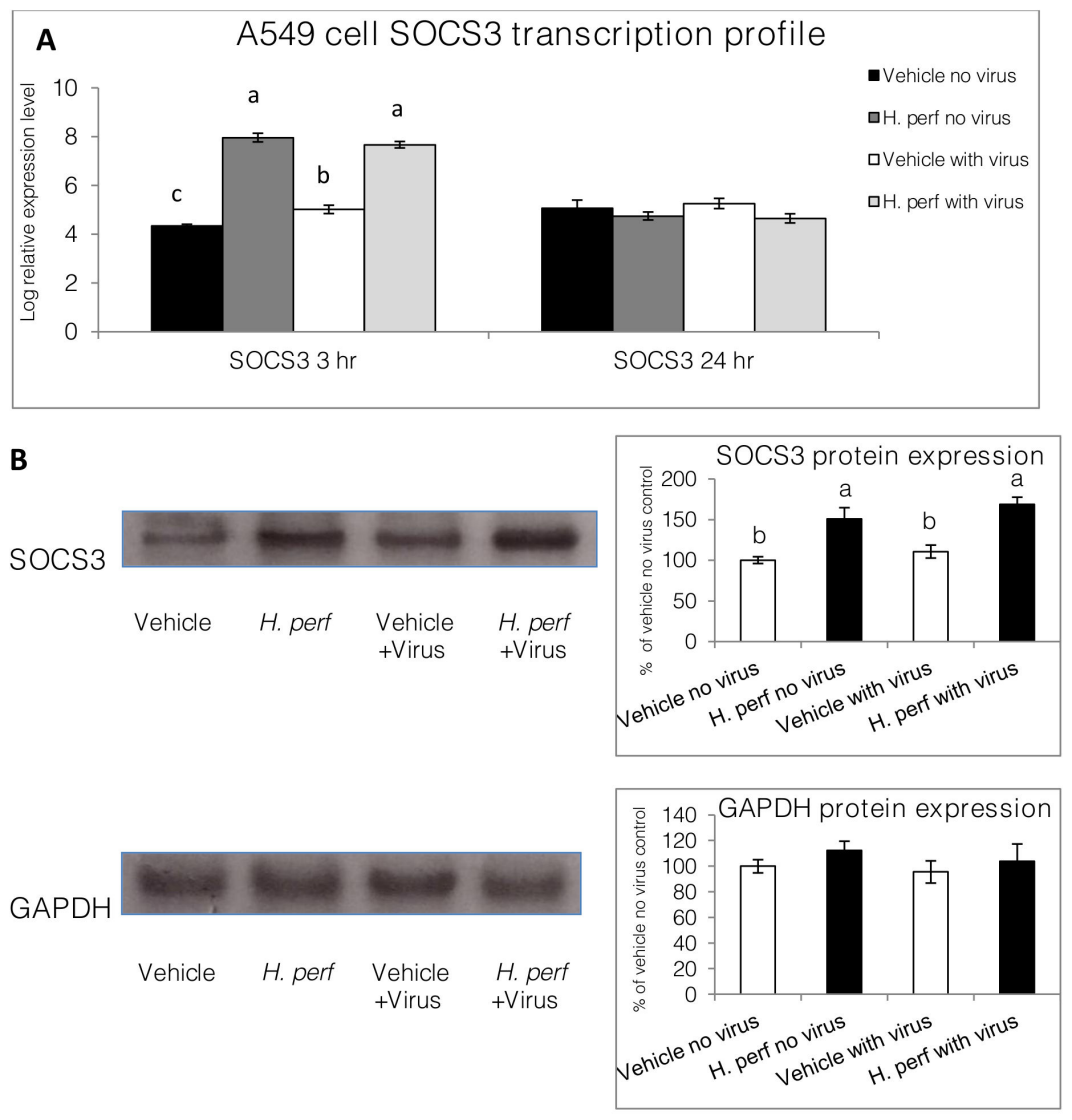
SOCS3 expression in A549. A549 cells were treated with DMSO vehicle control or 30 µg/mL *H*. *perforatum* extract in DMSO, with or without inoculation of H1N1 influenza virus. SOCS3 mRNA expression levels relative to and GAPDH (control) 3 hrs and 24 hrs after treatment are shown as log relative transcription level (Panel A. Mean ± SEM, N=3). Protein expression of SOCS3 24 hrs after the treatment was measured using Western blot and shown in Panel B (Mean ± SEM, N=3) as percentage of vehicle control without virus, together with a representative image. Values at the same time point are differentiated by individual letter labels when significant differences exist (p<0.05, a > b > c).

SOCS3 gene transcription was measured in A549 bronchial epithelial cells under the treatments of DMSO vehicle or 30 µg/mL H*. perforatum* extract, with and without H1N1 virus infection. After 3 hrs of treatment, H1N1 influenza virus significantly elevated SOCS3 transcription level in the DMSO vehicle treated cells, the extract increased SOCS3 transcription with or without virus as indicated by Figure **2** **(Panel A**). SOCS3, relative to GAPDH housekeeping reference, was not changed by either the treatments or virus infection at the 24 hr time point. At protein level, SOCS3 expression was also dramatically elevated by the *H* *. perf* extract treatment, while no significant increase was observed after virus challenge (**Panel B**). GAPDH protein expression remained unchanged in A549 cells under viral challenge or treatment.

### In vivo toxicity of *H* *. perforatum* extract

Mice gavaged with 5% ethanol vehicle control, 60 mg/kg H*. perforatum* extract, 110 mg/kg extract, and 220 mg/kg extract were monitored over the 3 week treatment regimen for their body weights and daily food consumption. No difference in body weight or food intake was found between these groups, neither did they differ in liver, kidney, spleen, stomach or intestine weight at the end of the study (data not shown). No apparent skin lesion or behavior abnormity was observed in *H* *. perforatum* treated groups.

### Body weight, food and water disappearance, and illness score of mice in 6-day low viral titer (10^5.0^ EID_50_) inoculation study

**Figure 3:**
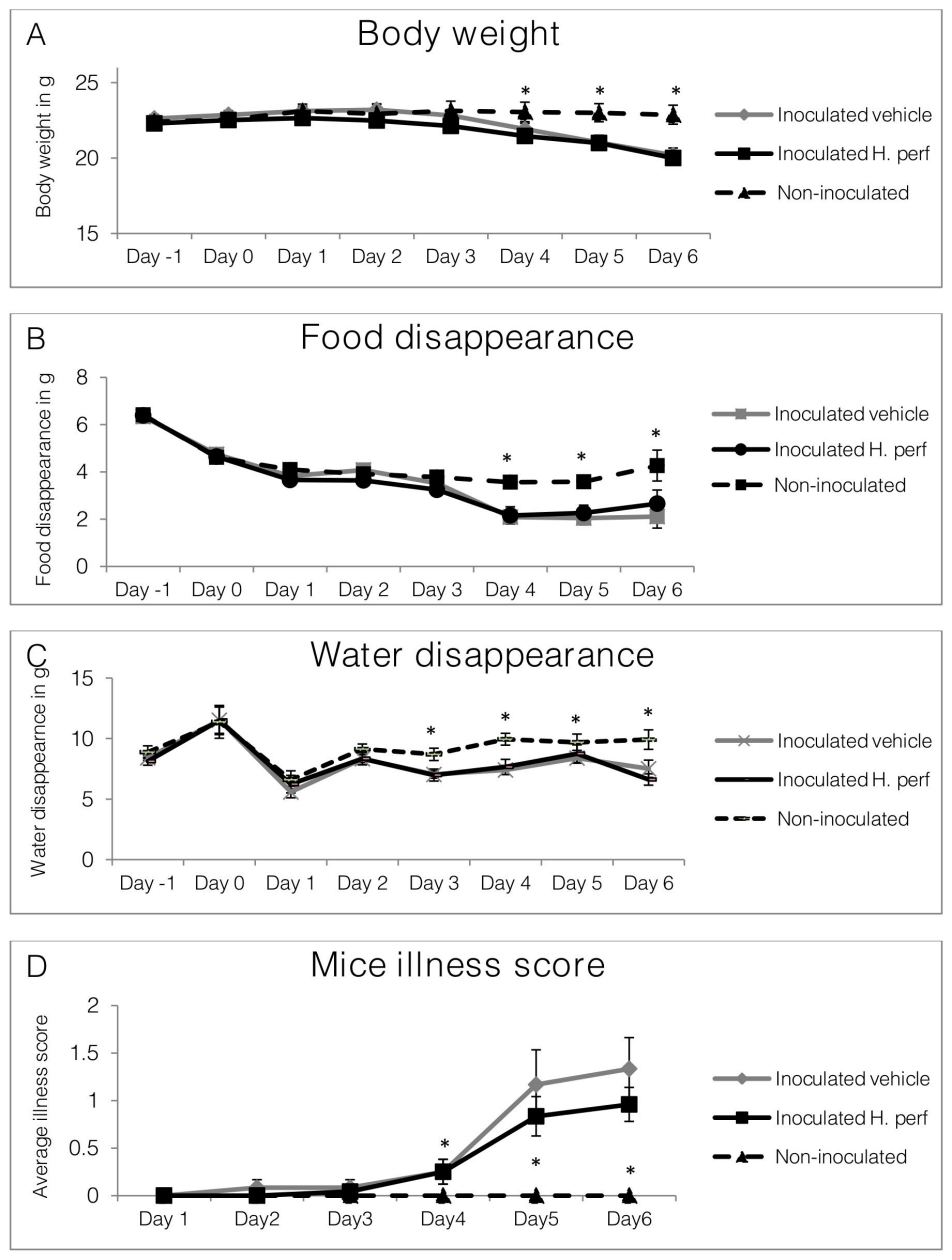
Body weight, food and water disappearance, and illness score of mice in 6-day low viral titer inoculation study. Mice were inoculated intranasally with H1N1 influenza virus (10^5.0^ EID_50_) on day 0 and gavaged with 5% ethanol vehicle control from day -1 to day 5 PI (N=12), or inoculated with virus on day 0 and gavaged with *H*. *perforatum* extract in 5% ethanol (N=12) at the rate of 110 mg/kg of body weight, or not inoculated with virus and gavaged with 5% ethanol (N=6). Daily records of body weight (A), food (B) and water disappearance (C), as well as mouse illness score (D) are shown as Mean ± SEM. Significant difference between non-inoculated and inoculated groups is labeled with * (p<0.05), while difference between inoculated vehicle and inoculated *H*. *perforatum* extract treated group is highlighted with # (p<0.05).

Daily records of body weight (Figure **3**, **panel A**), food (Figure **3**, **panel B**) and water (Figure **3**, **panel C**) disappearance are shown from day -1 to day 6 PI, while mouse illness scores are shown from day 1 to day 6 PI (**panel D**). Body weight of virus inoculated mice started to drop on day 3 and became significantly lower than the non-inoculated group by day 4. By day 6 PI, the discrepancy between the inoculated and non-inoculated mice was ~2.7 g or ~12% initial body weight. Food and water consumption, measured by daily food and water disappearance, had similar trends as body weight, with differences between inoculated and non-inoculated mice becoming significant after day 4 and day 3 PI, respectively. Inoculated mice treated with vehicle and *H* *. perforatum* extract had the same body weight, food and water intake over the course of the study. Illness score remained 0 during the study in the non-inoculated group, while the scores for the inoculated groups became significantly higher than 0 at day 4. The *H* *. perforatum* treated group had a lower illness score than the vehicle control treated group, although the difference was not statistically significant, likely due to high variability (p=0.09).

### Lung index and viral titer in 6-day low viral titer (10^5.0^ EID_50_) inoculation study

**Figure 4:**
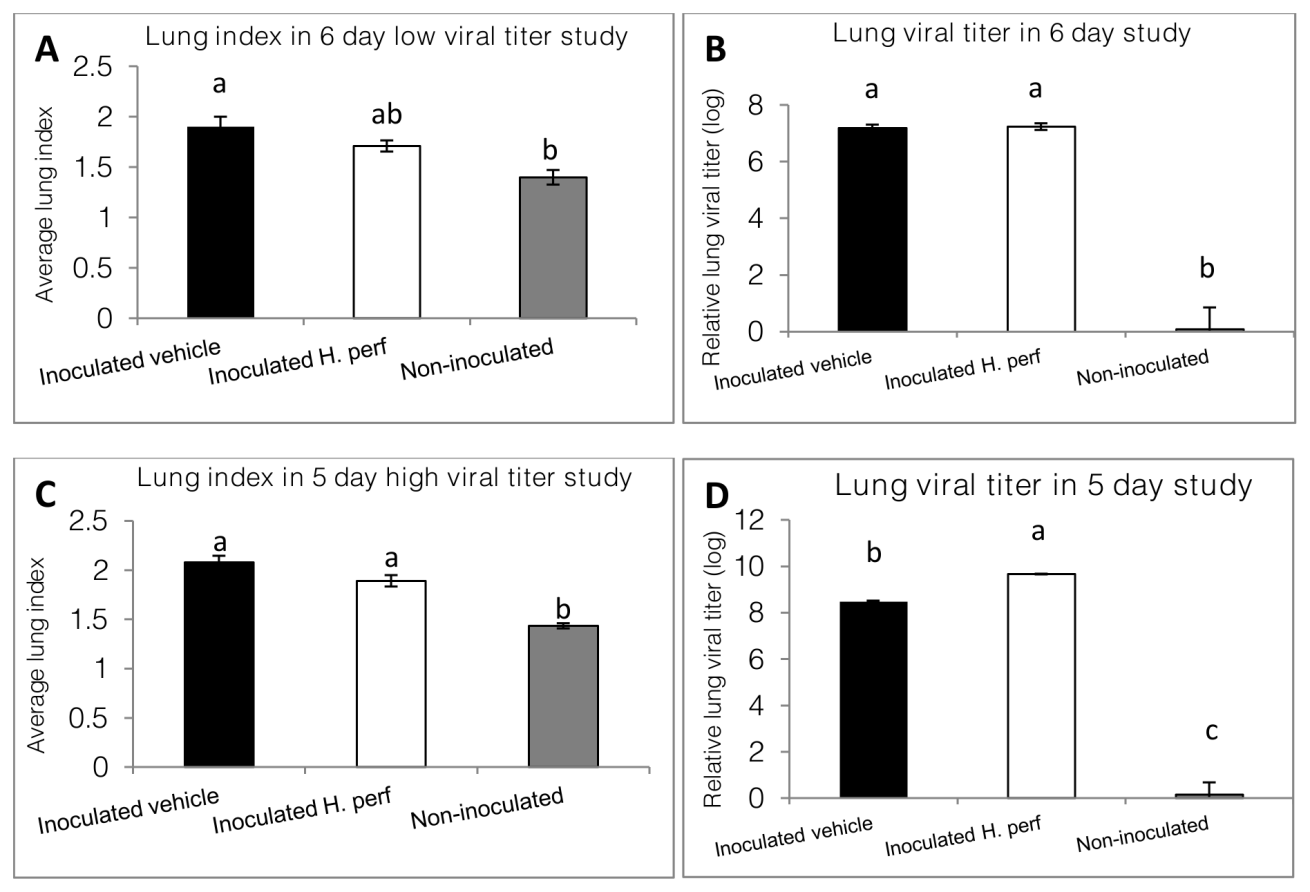
Lung index and viral titer of mice. Mice were inoculated intranasally with 10^5.0^ and 10^7.9^ EID_50_ of H1N1 influenza virus respectively on day 0 and gavaged with 5% ethanol vehicle control from day -1 to day 6 (N=12) or day 5 PI (N=15), or inoculated with virus on day 0 and gavaged with 110 mg/kg *H*. *perforatum* extract in 5% ethanol (N=12 for low viral titer study and N=15 for high viral titer study), or not inoculated with virus and gavaged with 5% ethanol (N=6 for 6-day study and N=5 for 5-day study). At the end of the study, the mouse lungs were weighed and used to calculate lung index (A and C) by histopathology, shown as Mean ± SEM. Lung viral titer (B and D) was measured using qRT-PCR, with log values for relative viral titer shown as Mean ± SEM. Values without the same label are different from each other statistically (p<0.05, a > b > c).

The lung index of the mice at the end of the study are shown in Figure **4** **, panel A**. The lung index of the inoculated vehicle group was significantly higher than that of the non-inoculated group, while the *H* *. perforatum* treated group was not significantly different from either group. Lung viral titer was measured for each mouse. The results, depicted in **panel B**, indicated relatively higher lung viral titer in the two inoculated groups, with no significant difference between them.

### Serum inflammatory cytokines in 6-day low viral titer (10^5.0^ EID_50_) inoculation study

Inflammatory cytokines IL-6 and IL-1β in mouse serum were measured using ELISA (Figure **S2**). IL-6 was elevated by H1N1 influenza viral inoculation and the *H* *. perforatum* treated group had a significantly lower IL-6 level in comparison to the inoculated vehicle group. No difference was found between the three groups regarding serum IL-1β level.

### BAL inflammatory cytokines and NO in 6-day low viral titer (10^5.0^ EID_50_) inoculation study

BAL samples collected from the mice were subject to multiplex analysis, and the results are shown in Table **1**. All cytokines and NO in the panel, except for eotaxin, IL-1α, IL-2, IL-3, IL-7, IL-9, and IL12 (p40), were significantly elevated after influenza virus inoculation. IL-2 was slightly decreased after inoculation although its level was very low (

< 2 pg/mL). IL-6 was higher in the *H*. *perforatum* extract treated group compared to the inoculated vehicle treated group. The two inoculated groups did not differ from each other in other cytokines or NO.

**Table 1:** Mouse BAL cytokine and NO levels in 6-day low viral titer (10^5.0^ EID_50_) inoculation study.

**Cytokines**	**Inoculated vehicle** (N=12)			**Inoculated *H* *. perf* ** (N=12)			**Non-inoculated** (N=6)			**Statistical effect**	
										**Infection**	*** H. perf ***
**Eotaxin**	**32.1**	±	5.6	**34.1**	±	5.2	**13.6**	±	11.4		
**G-CSF**	**985.7**	±	186.4	**937.6**	±	148.8	**1.2**	±	0.3	*	
**GM-CSF**	**18.2**	±	2.4	**15.3**	±	2.1	**1.3**	±	1.3	*	
**IFN-γ**	**2626.7**	±	687.2	**2401.6**	±	504.1	**0.8**	±	0.4	*	
**IL-1a**	**11.5**	±	1.4	**11.0**	±	1.7	**7.5**	±	2.7		
**IL-1b**	**10.1**	±	0.8	**10.2**	±	1.4	**2.4**	±	0.7	*	
**IL-2**	**1.5**	±	0.1	**1.4**	±	0.1	**2.5**	±	0.5	*	
**IL-3**	**1.4**	±	0.1	**1.4**	±	0.1	**1.0**	±	0.1		
**IL-4**	**0.8**	±	0.1	**0.8**	±	0.1	**0.4**	±	0.0	*	
**IL-5**	**15.5**	±	3.8	**16.1**	±	3.2	**0.2**	±	0.1	*	
**IL-6**	**933.7**	±	167.0	**1424.6**	±	305.9	**2.7**	±	1.3	*	**#**
**IL-7**	**0.5**	±	0.1	**0.5**	±	0.2	**0.3**	±	0.2		
**IL-9**	**98.9**	±	11.8	**100.2**	±	12.2	**69.8**	±	10.4		
**IL-10**	**159.4**	±	48.0	**128.1**	±	25.6	**0.8**	±	0.3	*	
**IL12(p40)**	**9.0**	±	1.0	**7.6**	±	1.5	**11.0**	±	2.9		
**IL-12(p70)**	**10.2**	±	1.2	**10.0**	±	1.4	**0.0**	±	0.0	*	
**IL-13**	**23.6**	±	2.6	**21.2**	±	4.2	**0.3**	±	0.2	*	
**IL-15**	**8.2**	±	1.1	**6.5**	±	1.4	**1.1**	±	0.6	*	
**IL-17**	**2.1**	±	0.2	**2.1**	±	0.3	**0.6**	±	0.2	*	
**IP-10**	**2348.4**	±	0.3	**2369.3**	±	0.4	**5.8**	±	2.5	*	
**KC**	**390.6**	±	38.2	**353.0**	±	67.5	**14.5**	±	2.7	*	
**LIF**	**87.3**	±	15.4	**82.9**	±	15.5	**0.4**	±	0.1	*	
**LIX**	**32.3**	±	9.2	**20.9**	±	8.4	**4.4**	±	4.4	*	
**MCP-1**	**425.1**	±	100.6	**426.4**	±	78.5	**1.0**	±	0.7	*	
**M-CSF**	**9.1**	±	1.0	**7.6**	±	0.8	**0.9**	±	0.6	*	
**MIG**	**11271**	±	1444	**11140**	±	1958	**8.8**	±	2.4	*	
**MIP-1a**	**34.5**	±	3.7	**33.0**	±	4.3	**4.8**	±	1.6	*	
**MIP-1b**	**302.7**	±	55.0	**299.8**	±	54.8	**1.8**	±	0.9	*	
**MIP-2**	**75.6**	±	11.2	**61.2**	±	4.7	**3.2**	±	2.0	*	
**RANTES**	**15.3**	±	1.7	**16.1**	±	2.4	**1.7**	±	0.2	*	
**TNF-α**	**10.7**	±	1.6	**10.6**	±	1.7	**1.4**	±	0.1	*	
**VEGF**	**23.7**	±	3.3	**18.5**	±	2.4	**6.7**	±	1.2	*	
**NO^1^**	**1.5**	±	0.3	**1.2**	±	0.2	**0.3**	±	0.1	*	

Data shown in pg/mL as Mean ± SEM (^1^NO unit is µM). Significant difference between non-inoculated and inoculated groups is labeled with * (p<0.05), while difference between inoculated vehicle control and inoculated *H* *. perforatum* extract treated group is highlighted with # (p<0.05).

### BAL cell population in 6-day low viral titer (10^5.0^ EID_50_) inoculation study

**Figure 5:**
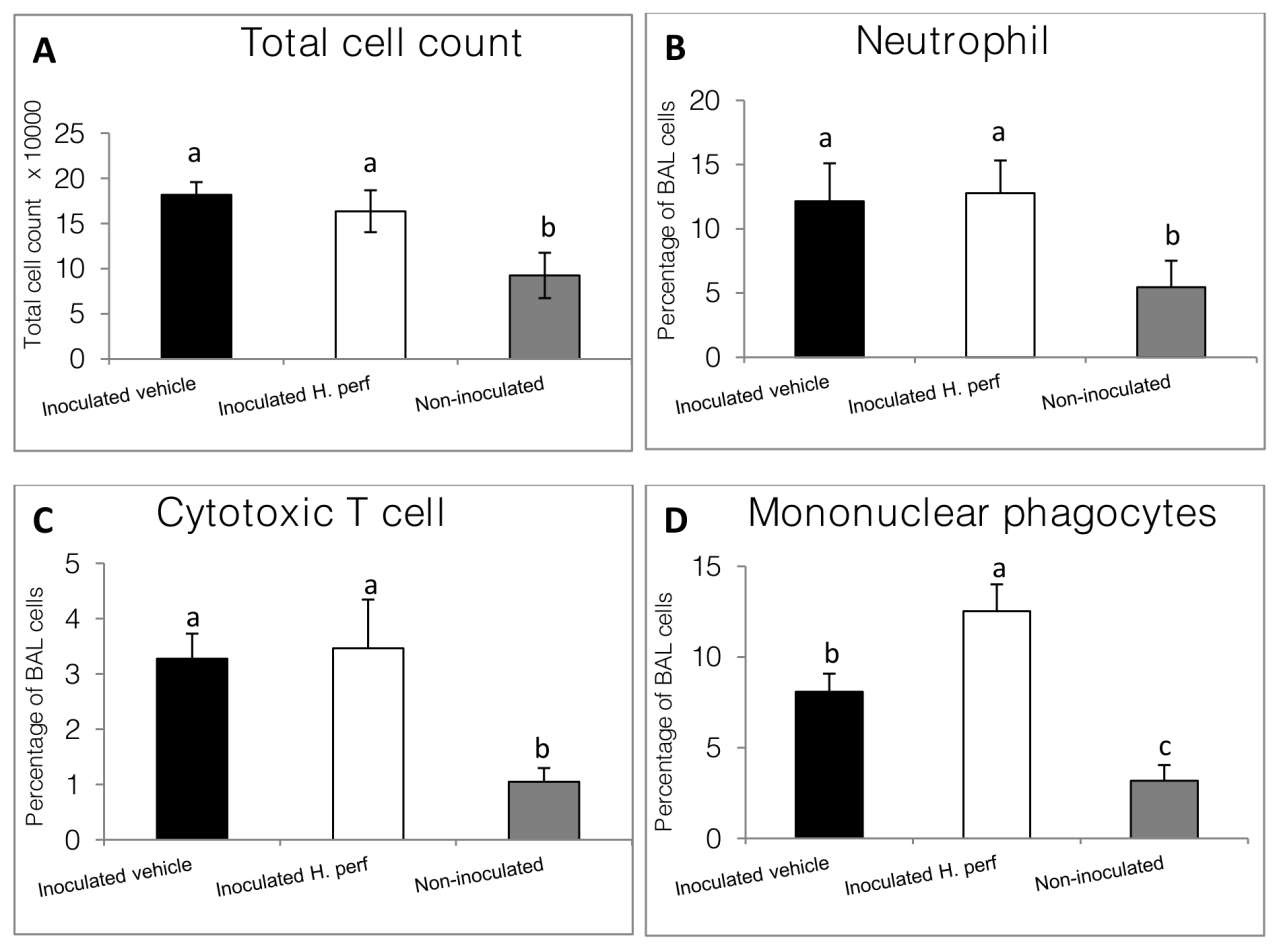
Mouse BAL cell population in 6-day low viral titer inoculation study. Mice were inoculated intranasally with H1N1 influenza virus (10^5.0^ EID_50_/mouse) on day 0 and gavaged with 5% ethanol vehicle control from day -1 to day 5 PI (N=12), or inoculated with virus on day 0 and gavaged with 110 mg/kg *H*. *perforatum* extract (N=12), or not inoculated with virus and gavaged with 5% ethanol (N=6). BAL cells were sorted using flow-cytometry. Total cell counts (A), and percentage of BAL cells being neutrophils (CD11b^+^ GR1^+^ high SSC)(B), cytotoxic T cells (CD8b^+^ low SSC)(C) and mononuclear phagocytes (GR1^int^ low SSC)(D) are shown as Mean ± SEM for each treatment group. Values without same label are different from each other statistically (p<0.05, a > b > c).

Cells in the BAL were analyzed using flow cytometry. Figure **5** shows the total cell counts (**panel A**) and percentage of neutrophils (**panel B**), CTLs (**panel C**), as well as mononuclear phagocytes (**panel D**) in the BAL. While inoculation with influenza virus increased the total number of cells in the BAL, the *H* *. perforatum* treated mice had somewhat lower total cell count than the vehicle treated mice, although not statistically significant. The percentages of neutrophils, mononuclear phagocytes, and CTLs increased after virus inoculation when compared to the non-inoculated mice. *H* *. perforatum* treatment led to higher percentage and total count (1.5×10^4^ vs. 2.1×10^4^) of pro-inflammatory mononuclear phagocytes, when compared to vehicle treated mouse after infection.

### Body weight, food and water disappearance, and illness score in 5-day high viral titer (10^7.9^ EID_50_) inoculation study

**Figure 6:**
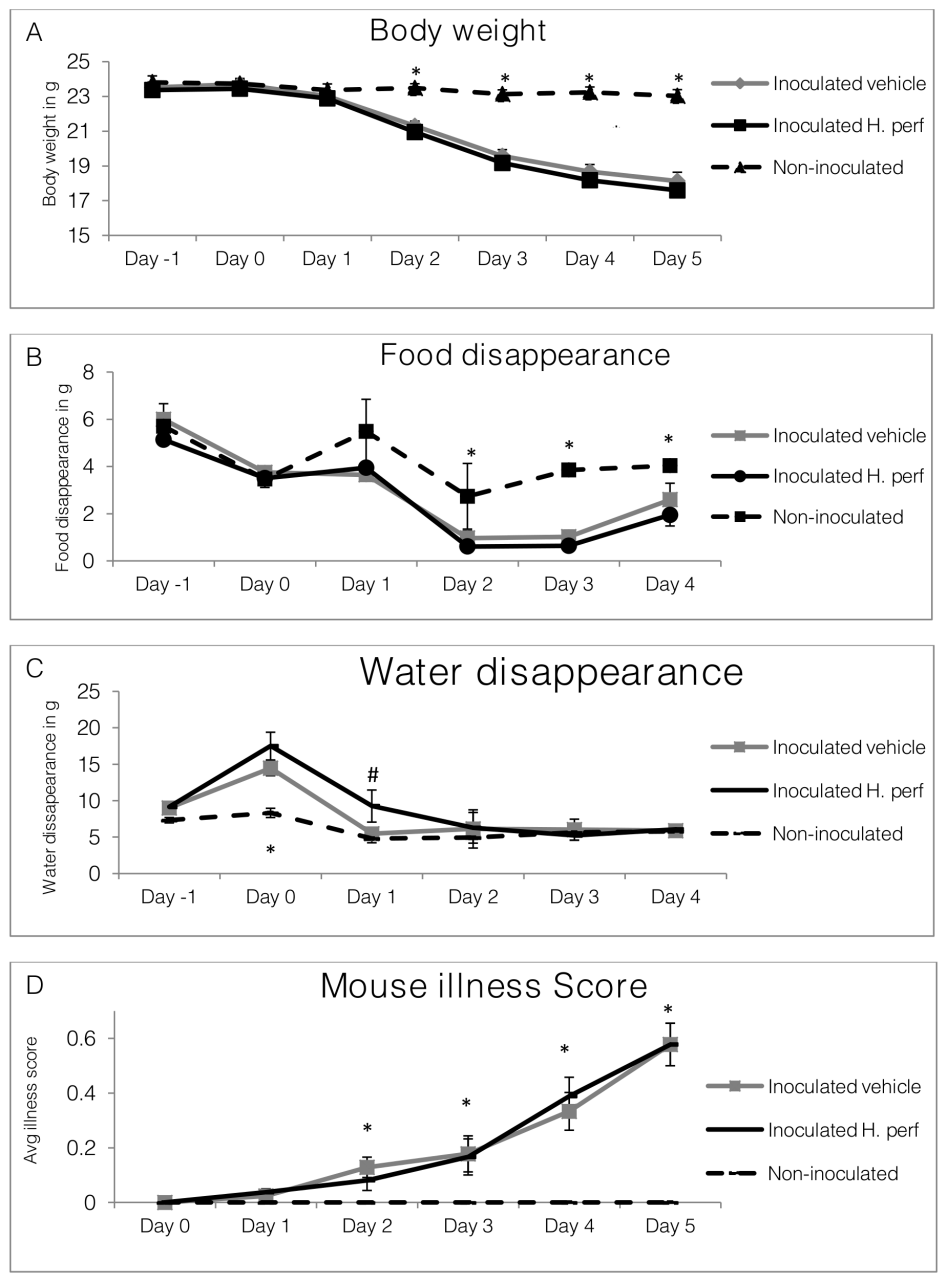
Body weight, food and water disappearance, and illness score of mice in 5-day high viral titer inoculation study. Mice were inoculated intranasally with H1N1 influenza virus (10^7.9^ EID_50_) on day 0 and gavaged with 5% ethanol vehicle control from day -1 to day 4 PI (N=15), or inoculated with virus on day 0 and gavaged with 110 mg/kg *H*. *perforatum* extract (N=15), or not inoculated with virus and gavaged with 5% ethanol (N=6). Daily records of body weight (from day -1 to day 5)(A), food (B) and water disappearance (C)(from day -1 to day 4), as well as mouse illness score (D)(from day 1 to day 5) are shown as Mean ± SEM. Significant difference between non-inoculated and inoculated groups is labeled with * (p<0.05), while difference between inoculated vehicle and inoculated *H*. *perforatum* group is highlighted with # (p<0.05).

Aiming to study the effect of *H* *. perforatum* on mice with severe influenza symptoms, a high viral dose of 10^7.9^ EID_50_ was used to inoculate the animals in a 5-day infection study. Daily body weight, food and water intake, and illness score measurements throughout the study are shown in Figure **6**. Due to the higher viral dose, significant body weight drop appeared just 2 days after inoculation and resulted in over 20% (5 g) weight lost at the end of the 5 day study (**panel A**). However, the two inoculated groups were not significantly different in body weight. Records of food (**panel B**) and water (**panel C**) disappearance on day 5 PI were not collected because moistened diets were given in petri dishes to the animals that had lost over 15% of body weight to improve access to food and water. Food disappearance was lower in the inoculated groups from days 2 through 4 PI. Water disappearance was similar throughout the study between the groups. More water disappeared in the virus-inoculated groups on day 0 in comparison to the non-inoculated group. At the same time, the *H* *. perforatum* treated group had greater water disappearance than the virus-inoculated mice treated with vehicle on day 1 PI. Mouse illness scores for the virus-inoculated groups were significantly higher than that of the non-inoculated group from day 2 through day 5 PI, with no difference between mice treated with ethanol vehicle and *H* *. perforatum* extract (**panel D**).

### Lung index and viral titer in 5-day high viral titer (10^7.9^ EID_50_) inoculation study

Lung index and Influenza A H1N1 viral titer for each mouse were measured at the end of the 5 day study and are shown in Figure **4**. Both lung index (**panel C**) and viral titer (**panel D**) of the mice challenged with influenza virus were elevated comparing to the non-challenged group. Mice treated with *H* *. perforatum* extract had significantly higher viral titer than that of the vehicle treated animals after Influenza A H1N1 inoculation, while the lung index was the same in the two inoculated groups, both were higher than that in the non-inoculated group.

### BAL inflammatory cytokines and NO in 5-day high viral titer (10^7.9^ EID_50_) inoculation study

Table **2** demonstrates the cytokines and NO levels in lung BAL measured using multiplex. Except for IL-9, IL12 (p40), and LIX, all cytokines plus NO were significantly elevated after influenza virus challenge. *H* *. perforatum* treatment resulted in higher levels of a variety of inflammatory cytokine and NO when compared to the inoculated vehicle treated control. Cytokines increased by *H* *. perforatum* treatment included eotaxin, G-CSF, GM-CSF, M-CSF, IL-6, IL-12(p70), IL-13, IL-15, LIF, MCP-1, and MIP-2. At the same time, IP-10 was found to be reduced by *H* *. perforatum* treatment.

**Table 2:** Mouse BAL cytokine and NO levels in 5-day high viral titer inoculation study (10^7.9^ EID_50_).

**Cytokines**	**Inoculated vehicle** (N=15)			**Inoculated *H* *. perf* ** (N=15)			**Non-inoculated** (N=6)			**Statistical effect**	
										**Infection**	*** H. perf ***
**Eotaxin**	**110.2**	±	23.9	**194.9**	±	36.7	**7.0**	±	2.0	*	**#**
**G-CSF**	**3.2**	±	1.1	**8.5**	±	2.1	**0.0**	±	0.0	*	**#**
**GM-CSF**	**21.4**	±	2.3	**29.2**	±	2.1	**0.8**	±	0.8	*	**#**
**IFN-γ**	**497.4**	±	212.1	**378.8**	±	141.2	**0.4**	±	0.3	*	
**IL-1a**	**13.1**	±	2.1	**19.2**	±	2.3	**3.6**	±	2.3	*	
**IL-1b**	**10.1**	±	1.0	**8.2**	±	0.4	**3.0**	±	0.4	*	
**IL-2**	**1.5**	±	0.1	**1.4**	±	0.1	**2.2**	±	0.3	*	
**IL-3**	**1.7**	±	0.1	**2.0**	±	0.2	**1.0**	±	0.1	*	
**IL-4**	**0.8**	±	0.1	**0.9**	±	0.1	**0.4**	±	0.0	*	
**IL-5**	**26.2**	±	7.8	**39.3**	±	10.1	**0.1**	±	0.0	*	
**IL-6**	**942.8**	±	205.6	**1667.8**	±	301.9	**0.3**	±	0.2	*	**#**
**IL-7**	**0.7**	±	0.1	**0.8**	±	0.1	**0.2**	±	0.1	*	
**IL-9**	**114.2**	±	15.3	**120.0**	±	17.3	**85.1**	±	18.3		
**IL-10**	**75.2**	±	42.3	**35.8**	±	8.3	**0.1**	±	0.1	*	
**IL12(p40)**	**6.7**	±	1.3	**8.3**	±	1.4	**3.3**	±	2.0		
**IL-12(p70)**	**10.6**	±	1.9	**16.4**	±	2.2	**0.1**	±	0.1	*	**#**
**IL-13**	**22.9**	±	4.7	**42.2**	±	5.8	**0.0**	±	0.0	*	**#**
**IL-15**	**8.5**	±	1.0	**13.3**	±	2.0	**1.1**	±	0.5	*	**#**
**IL-17**	**2.1**	±	0.3	**2.4**	±	0.3	**0.5**	±	0.1	*	
**IP-10**	**2434.6**	±	248.2	**1614.9**	±	98.8	**4.5**	±	0.7	*	˅
**KC**	**150.7**	±	39.6	**176.5**	±	24.2	**11.3**	±	1.0	*	
**LIF**	**119.2**	±	24.3	**420.2**	±	98.4	**0.6**	±	0.1	*	**#**
**LIX**	**172.4**	±	129.6	**3.2**	±	1.8	**0.0**	±	0.0		
**MCP-1**	**817.8**	±	188.1	**1260.4**	±	174.0	**0.3**	±	0.3	*	**#**
**M-CSF**	**11.0**	±	1.5	**16.1**	±	1.8	**0.5**	±	0.4	*	**#**
**MIG**	**8644.9**	±	2158	**10772**	±	2203	**8.2**	±	2.4	*	
**MIP-1a**	**32.1**	±	5.7	**31.6**	±	0.0	**5.7**	±	1.7	*	
**MIP-1b**	**131.8**	±	30.7	**149.4**	±	26.2	**1.4**	±	0.7	*	
**MIP-2**	**89.7**	±	11.8	**136.1**	±	14.1	**3.8**	±	1.9	*	**#**
**RANTES**	**15.7**	±	3.0	**19.5**	±	1.9	**1.3**	±	0.1	*	
**TNF-α**	**8.2**	±	1.6	**9.9**	±	1.2	**1.3**	±	0.1	*	
**VEGF**	**33.8**	±	8.7	**30.2**	±	4.0	**8.1**	±	1.2	*	
**NO^1^**	**3.8**	±	0.5	**5.1**	±	0.3	**1.4**	±	0.6	*	**#**

Data shown in pg/mL as Mean ± SEM (^1^NO unit is µM). Significant difference between non-inoculated and inoculated groups is labeled with * (p<0.05), while difference between inoculated vehicle control and inoculated *H* *. perforatum* extract treated group is highlighted with # (increased in *H* *. perforatum* treated group) and ˅ (decreased in *H* *. perforatum* treated group) (p<0.05).

### BAL cell population in 5-day high viral titer (10^7.9^ EID_50_) inoculation study

**Figure 7:**
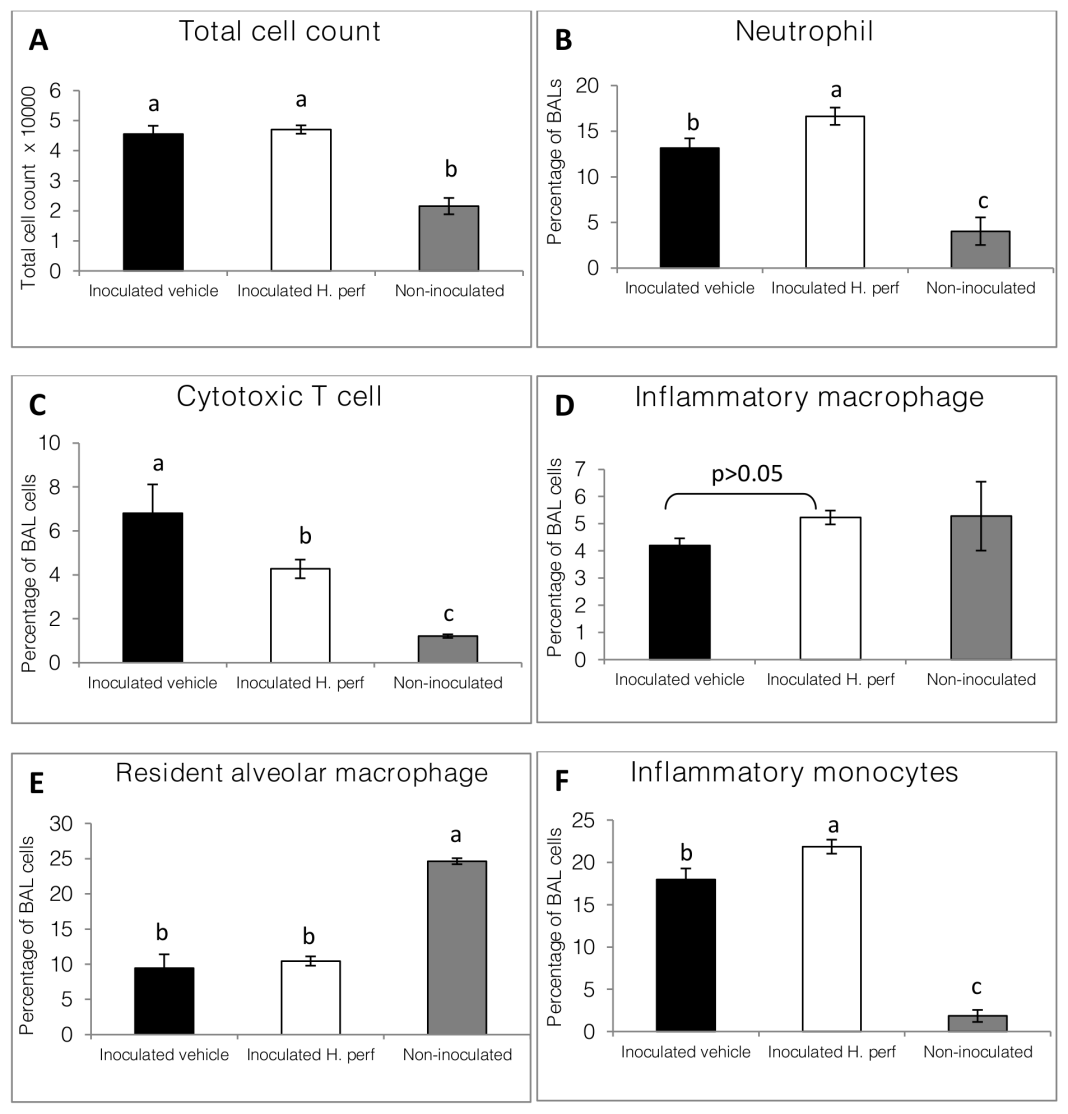
Mouse BAL cell population in 5-day high viral titer inoculation study. Mice were intranasally inoculated with H1N1 (10^7.9^ EID_50_) influenza virus on day 0 and gavaged with 5% ethanol vehicle control from day -1 to day 4 PI (N=15), or inoculated with virus on day 0 and gavaged with 110 mg/kg *H*. *perforatum* extract (N=15), or not inoculated with virus and gavaged with 5% ethanol (N=6). BAL cells were subject to flow-cytometry. Total cell counts (A), and percentage of BAL cells being neutrophils (CD11b^+^ GR1^+^ high SSC)(B), cytotoxic T cells (CD8b^+^ low SSC)(C), induced macrophages (CD11b^+^ GR1^-^ high auto-fluorescent)(D), resident alveolar macrophages (CD11b^-^ GR1^-^ high auto-fluorescent)(E) and inflammatory monocytes (CD11b^+^ GR1^int^ low SSC)(F) are shown as Mean ± SEM for each treatment group. Values without same over bar label are statistically different from each other (p<0.05, a > b > c).

Total BAL cell number (**panel A**) and neutrophil cell percentage (**panel B**) were significantly higher in the virus-inoculated groups than in the non-inoculated groups, as shown in Figure **7**. The *H* *. perforatum* treated group had more neutrophils and inflammatory monocytes (**panel F**) than the inoculated-vehicle group, with a lower percentage of cytotoxic T cells (**panel C**). Although no significant difference was found between all three groups in the percentage of inflammatory macrophages (**panel D**), the inoculated groups had higher numbers of these cells (1.9×10^3^ for vehicle and 2.5×10^3^for *H* *. perforatum* ) than the non-inoculated group (1.1×10^3^). The *H* *. perforatum* treated mice had a slightly higher number of inflammatory macrophages than the vehicle treated group, although the difference was not statistically significant. Resident macrophages accounted for a lower portion of BAL cells in the inoculated groups than in the non-inoculated group, but were not different between the two inoculated groups (**panel E**).

### Body weight, food and water consumption, and illness score in 10-day long term low viral titer (10^5.1^ EID_50_) inoculation study

**Figure 8:**
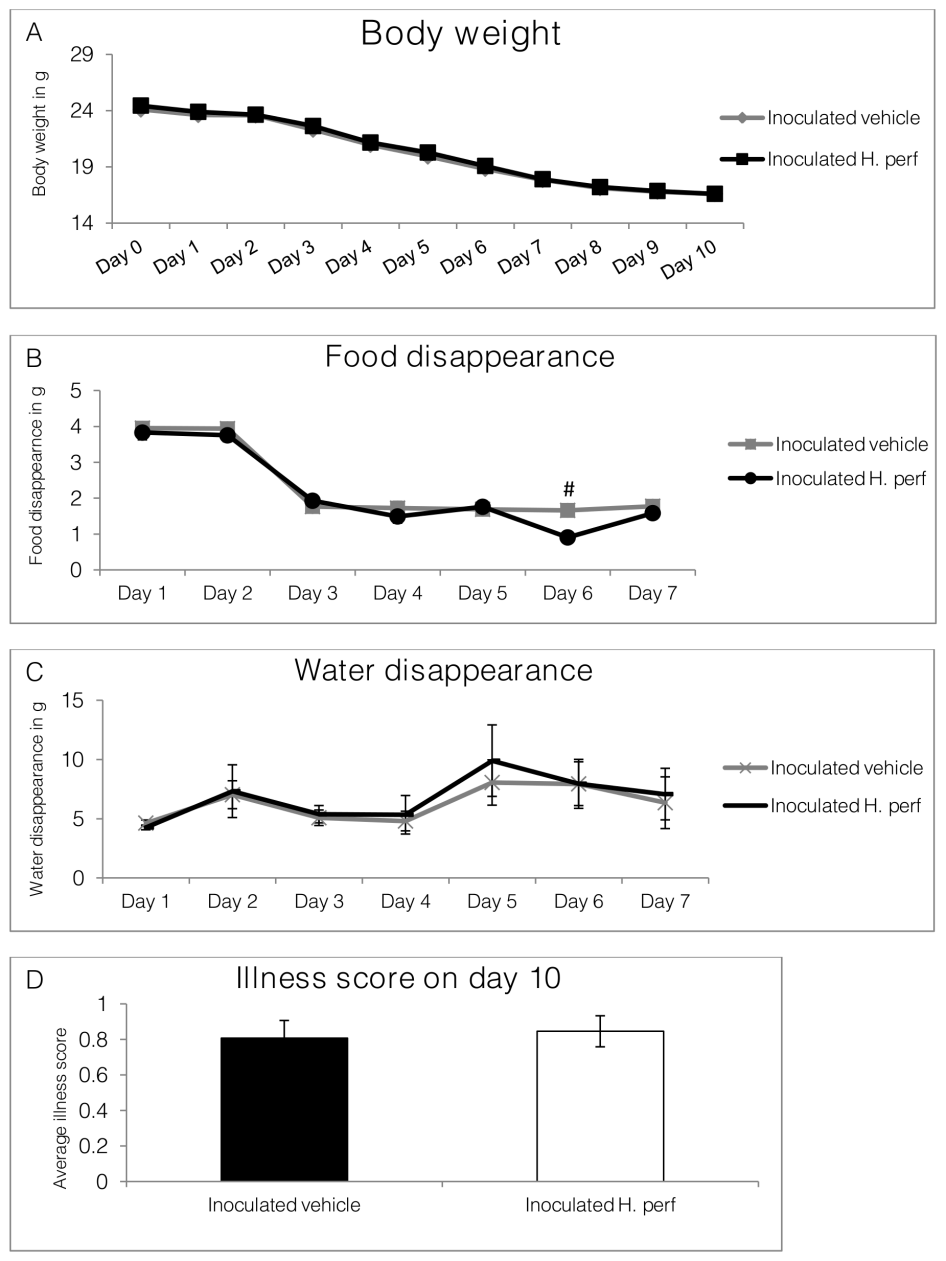
Body weight, food and water disappearance, and illness score of mice in 10-day long term low viral titer inoculation study. Mice were inoculated intranasally with H1N1 influenza virus (10^5.1^ EID50) on day 0 and gavaged with 5% ethanol vehicle control from day 5 to day 9 PI (N=13), or inoculated with virus (10^5.1^ EID_50_) on day 0 and gavaged with 110 mg/kg *H*. *perforatum* extract (N=13). Daily records of body weight (from day 0 to day 10), food and water disappearance (from day 1 to day 7), as well as mouse illness score on day 10, are shown as Mean ± SEM. Significant difference between inoculated vehicle and inoculated *H*. *perforatum* group is highlighted with # (p<0.05).

In order to study whether administrating *H* *. perforatum* extract during later phase of influenza virus inoculation could alleviate inflammatory lung lesion, a 10-day inoculation study was conducted, with gavage beginning at day 5 PI. Daily records of body weight (Figure **8** **, panel A**) from day 0 until day 10 PI, food (Figure **8** **, panel B**) and water (Figure **8** **, panel C**) disappearance are shown from day 0 to day 7 PI. The lack of diet and water disappearance data after day 7 PI was due to animals being provided these in petri dishes in the cage to allow access. Mouse illness score on day 10 PI is shown in **panel D**. Body weight of these inoculated mice began decreasing progressively by day 3 PI and body weight losses reached ~ 16 g on average at the end of the study. No difference in either endpoint, except for food intake on day 6 PI, was noted between the two treatments. *H* *. perforatum* treated mice consumed lower amount of diet on day 6 PI, but returned to the same level as the vehicle control group on day 7 PI.

### Lung lesion score and viral titer in 10-day long term low viral titer (10^5.1^ EID50) inoculation study

**Figure 9:**
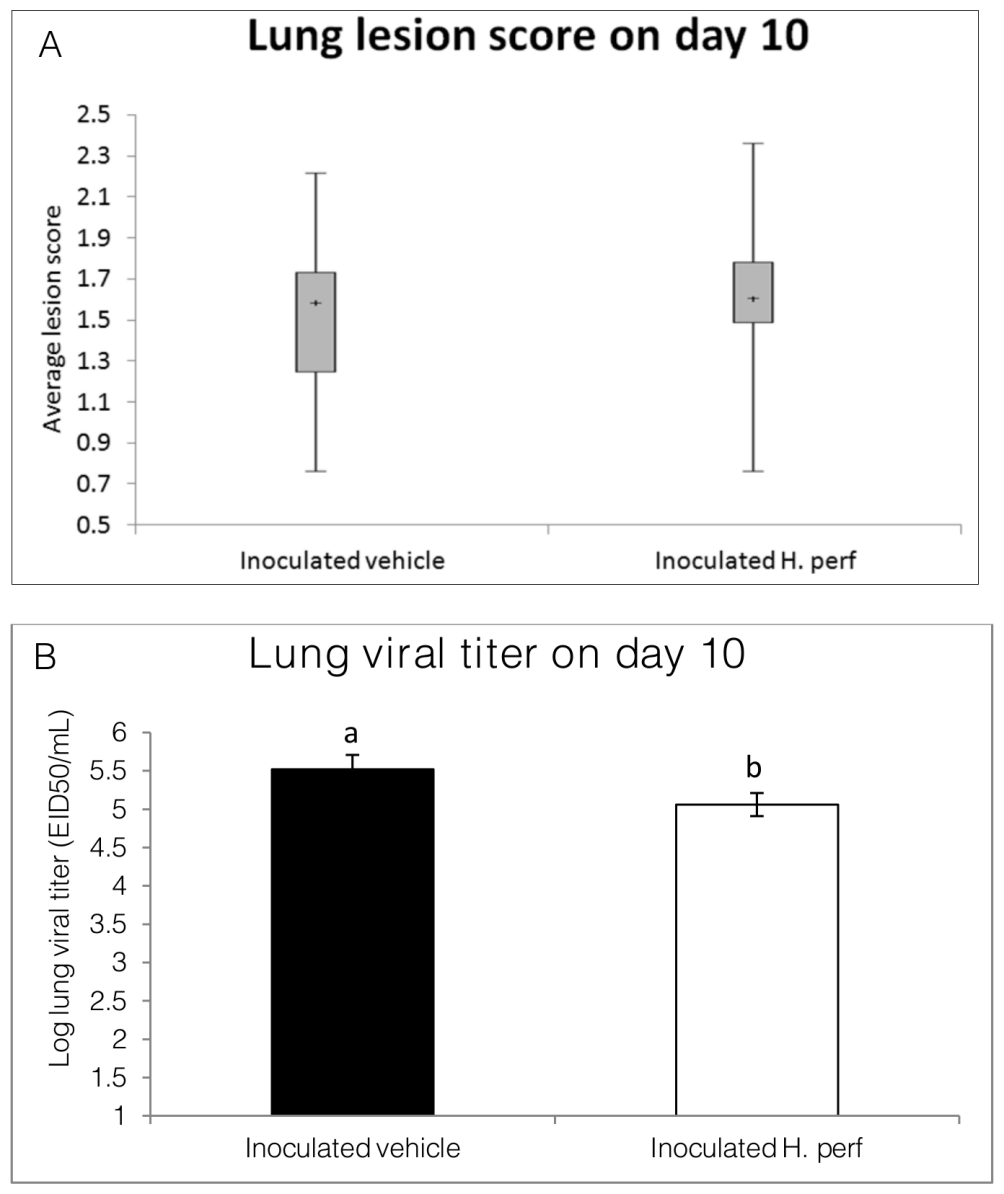
Lung lesion score and lung viral titer in 10-day long term low viral titer inoculation study. Mice were inoculated intranasally with H1N1 influenza virus (10^5.1^ EID50) on day 0 and gavaged with 5% ethanol vehicle control (N=13), or 110 mg/kg *H*. *perforatum* extract (N=13) from day 5 to day 9 PI. Microscopic lung lesion scores are shown in box plots. Lung viral titer was measured using qRT-PCR and shown as relative titer in log scale (Mean ± SEM). Values without same over bar label are statistically different from each other (p<0.05).

Lung lesion score and H1N1 viral titer for each mouse was measured at the end of the 10-day study and are shown in Figure **9**. Lung lesion score was not significantly different between the two groups, while lung viral titer of mice treated with *H* *. perforatum* extract was slightly lower than that of the vehicle group.

### Expression of SOCS3 gene in BALB/c mouse lung

**Figure 10:**
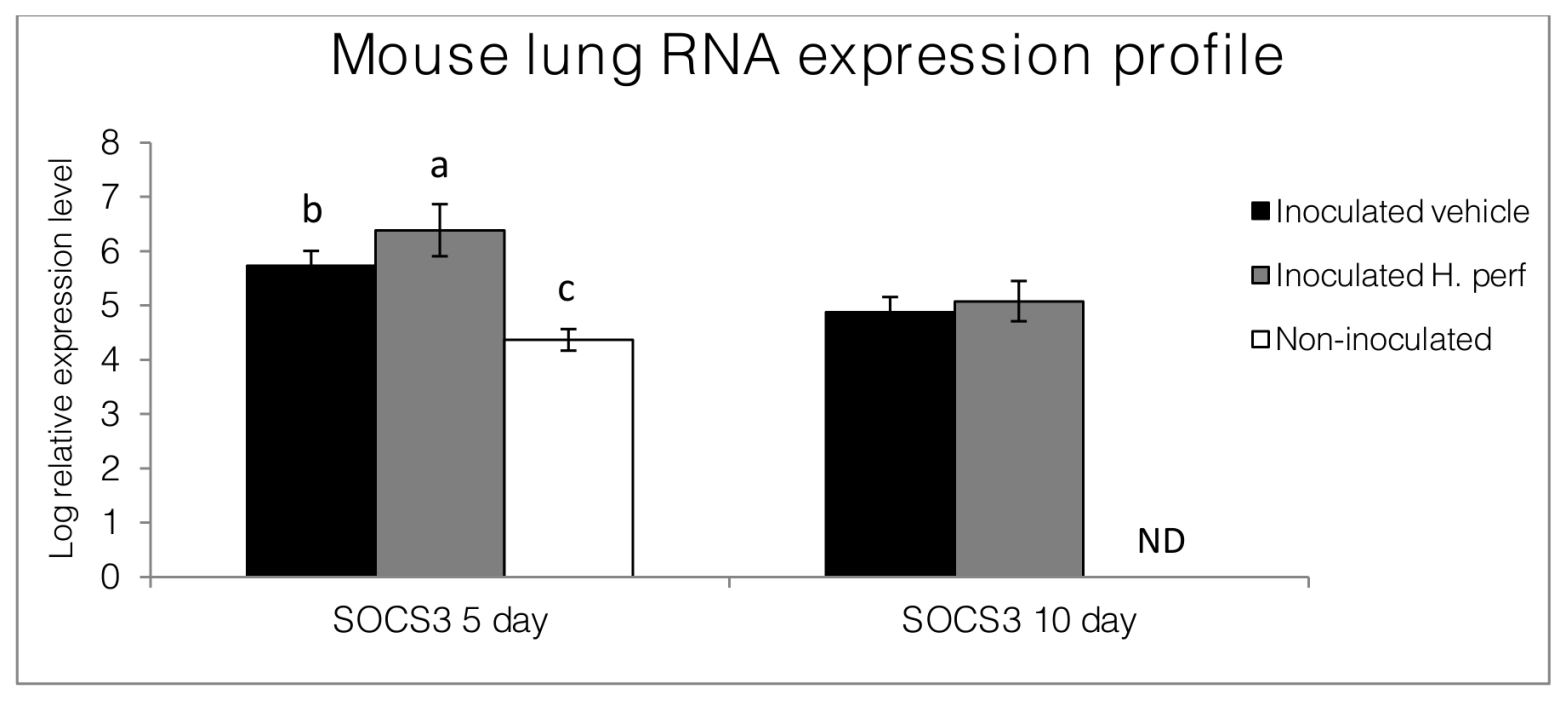
RNA transcription profiles the lung of BALB/c mice. Expression levels of SOCS3 relative to GAPDH in lungs collected at the end of the 5-day high viral titer inoculation study (10^7.9^ EID_50_, N=15 for inoculated groups and N=6 for non-inoculated group) and 10-day low viral tier inoculation study (10^5.1^ EID_50_, N=13 for each group) were measured and shown as log relative transcription level (Mean ± SEM). Values at the same time point are differentiated by individual over bar labels when significant differences were found (p<0.05). ND=not determined (no non-inoculated group in the 10 day study).

RNAs from lungs collected from the 5-day high viral titer and 10-day low viral titer studies were quantified for SOCS3 gene expression, as demonstrated in Figure **10**. SOCS3 transcription was elevated in the inoculated groups 5 days PI, and the *H* *. perforatum* treated mice showed higher level of SOCS3 mRNA compared to those receiving vehicle treatment. By day 10 PI, both inoculated groups had the same SOCS3 expression level in relation to the GAPDH housekeeping gene.

### Survival curve of mice treated with *H* *. perforatum* extract

**Figure 11:**
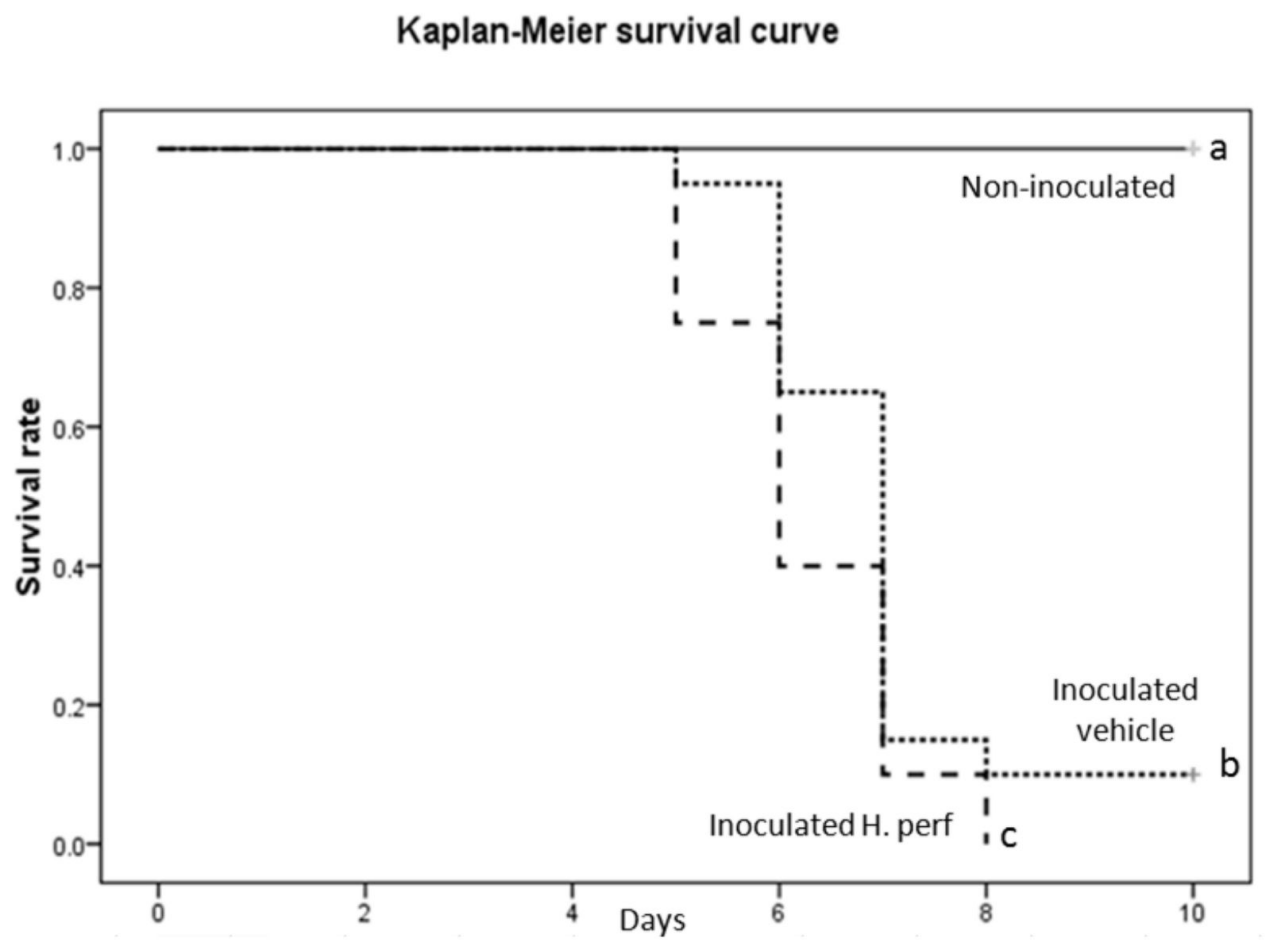
Survival curves of BALB/c mice inoculated with high viral titer. Kaplan-Meier survival curves of mice that received *H*. *perforatum* extract or 5% ethanol vehicle control, with or without intranasal inoculation of H1N1 influenza (10^7.9^ EID_50_) are shown (N=20 for each of the inoculated group, N=5 for the non-inoculated group). Curves with different letter annotation are significantly different from each other (P<0.05).

Mice inoculated with high viral titer (***10*^*7.9*^*EID*_*50*_**) of H1N1 virus received either 5% ethanol vehicle control or *H* *. perforatum* treatment. The survival curves under the two treatments are shown in Figure **11**. Although the body weight, food and water disappearance were not different between the two groups (data not shown), *H* *. perforatum* treatment led to earlier mortality as compared to the vehicle control, as indicated by the significant difference in the two survival curves. By day 8 PI, all mice in the *H* *. perforatum* group were dead, while 2 mice out of 20 in the vehicle group survived the entire 10-day observation period.

## Discussion

Our prior studies on the anti-inflammatory potential of *H* *. perforatum* were conducted using the RAW 264.7 mouse macrophage model [19], although these macrophages are important in responding to infectious agents, when it comes to influenza virus, respiratory epithelial cells are the target of infection and are the site at which the initiation of an immune response occurs [26]. MCP-1 and IP-10 are released by lung epithelial cells to recruit monocytes, NK cells and neutrophils, while IL-6 regulates the innate immune cell functions through the IL-6 receptor (IL-6R) [27]. In contrast to our prior observation in lipopolysaccharide (LPS)-induced macrophages, we found that the *H* *. perforatum* extract drastically increased IL-6 production by A549 cells, with or without virus stimulation (Figure **1**). LPS-induced IL-6 in macrophages was decreased by *H* *. perforatum* treatment in our previous study [28], and decreasing IL-6 was found to be important for the anti-depressive efficacy of *H* *. perforatum* treatment in rodents [29,30]. At the same time, influenza virus-induced TNF-α, MCP-1 and IP-10 production by epithelial cells were decreased by the extract, suggesting anti-inflammatory activity similar to what was observed in macrophages.

Influenza virus induced SOCS3 elevation in A549 cells shortly after inoculation has been reported by Pauli et al. and described as a mechanism through which the virus may compromise innate immune response [18,31]. We observed similar results in the current study, as H1N1 virus significantly increased SOCS3 gene transcription in A549 cells 3 hrs PI. *H* *. perforatum* extract treatment itself induced an even higher level of SOCS3 transcription in A549 cells, with or without influenza virus inoculation (Figure **2**). The concurrence of SOCS3 and IL-6 elevation is likely to be connected, as the IL-6R signaling depends on JAK-STAT and SOCS3 over-expression was shown to coincide with over-production of BAL IL-6 in mouse lung [32]. The divergent changes in different cytokines could be attributed to the dependence of IL-6 release on the activation of NF-κB, which was not inhibited by *H* *. perforatum* extract treatment, while IP-10 and MCP-1 production are activated by the IFN-γR/IL-6R-JAK-STAT pathway, which was inhibited by SOCS3. It should be noted that TNF-α, a primarily inflammatory cytokine produced by macrophages, NO-producing dendritic cells, and CD8+ T cells during influenza virus infection, was only released by epithelial cells at a very low level even with virus inoculation. Whether IL-6 over-production was directly induced by *H* *. perforatum* extract treatment or indirectly as a compensation for the interfered IL-6R signaling cascade remains unknown.

The animal studies provided information regarding the overall impact of *H* *. perforatum* extract treatment during influenza virus inoculation. When the viral titer of inoculum was low (10^5.0^ EID_50_) and illness was mild, no significant change in body weight and food and water consumption of mice was seen in the *H* *. perforatum* extract treated group (Figure **3**). BAL IL-6 was elevated by *H* *. perforatum* extract treatment (Table **1**), consistent with the A549 cell response described above. Because macrophage and monocyte infiltration usually reach peak levels between 5-10 days post inoculation of influenza virus, the higher percentage of BAL pulmonary mononuclear phagocytes found in *H* *. perforatum* extract treated mice may indicate a shift of the phagocyte exudation timeline or an overall larger recruited phagocyte population into the lung (Figure **5**) [33-35]. Overall, *H* *. perforatum* extract treatment did not ameliorate influenza virus infection when viral dose was at a level of 10^5.0^ EID_50_.

Our next effort was to increase viral dose to inflict more severe influenza conditions in mice in anticipation of being able to see more significant impact of the *H* *. perforatum* extract. Under a high viral titer of 10^7.9^ EID50 (>50% mortality dose), mice became sick earlier and demonstrated steeper body weight loss in the 5-day infection study (Figure **6**). Although there was a trend of lower body weight and food intake for the *H* *. perforatum* treated group, the 5-day duration was not long enough to show any significant effect of the treatment. Interestingly, lung viral titer was significantly higher in the mice that received *H* *. perforatum* extract, suggesting less efficient viral clearance (Figure **4**). The analysis of BAL cell populations at the end of the 5-day study indicated higher amount of pro-inflammatory neutrophils, macrophages, and monocytes, and lower amount of CD8β+ cells in the *H* *. perforatum* extract treatment group (Figure **7**). This profile, together with the corresponding increase of most major pro-inflammatory BAL cytokines, chemokines and NO, indicated more severe inflammation resulted from *H* *. perforatum* treatment (Table **2**). The only exception was the reduction of IP-10 level in the BAL that could have resulted from the lower number of T cells, which are known to stimulate IP-10 production through IFN-γ. This ‘pro-inflammatory’ phenomenon was contrary to the *in vitro* anti-inflammatory finding previously observed. A likely explanation is that an early innate-immune inhibitory effect of *H* *. perforatum* interfered with the necessary anti-viral host response. Lung SOCS3 expression was found to be increased after virus inoculation and further potentiated by the *H* *. perforatum* extract treatment. It is possible that the increased expression of SOCS3 by the extract inhibited innate immune response and delayed subsequent viral clearance by CTLs, resulting in higher viral titer by day 5 PI and more severe virus-induced inflammation, although the majority of CTLs usually enter the lung later than this time point [18]. Nonetheless, whether these changes in BAL reflected overall detrimental impact from the extract remains doubtful, especially given that neutrophils could ameliorate lung injury during influenza and monocyte/macrophages are important for anti-viral immunity [6,36]; and considering that that the timing of neutrophil or monocyte recruitment is probably important in determining whether increases in these cell populations is beneficial or detrimental. When the duration of study was extended, high viral titer inoculation (10^7.9^ EID_50_) combined with *H* *. perforatum* extract treatment led to earlier and steeper decline of survival curve (Figure **11**), indicating that the altered immune response could be detrimental to survival in severely infected animals.

During the early phase of influenza virus infection inflammation is critical for viral containment and immune activation [2]. But in the later phase, once the viral load has been significantly decreased, anti-inflammatory intervention may help reduce tissue damage. This hypothesis drove us to the 10-day low viral titer (10^5.1^ EID_50_) inoculation study, in which *H* *. perforatum* extract was not administrated until 5 days PI. The histopathological evaluation of lung tissues did not reveal any lung lesion improvement associated with *H* *. perforatum* extract treatment in the inoculated group. However, the viral titer was slightly lower in the *H* *. perforatum* extract treated group by day 10 PI, which could result from its anti-viral activity as reported by Liu et al. [25]. Other than that, our study failed to find any protective effect of late phase administrated *H* *. perforatum* extract against influenza virus induced inflammatory damage.

**Figure 12:**
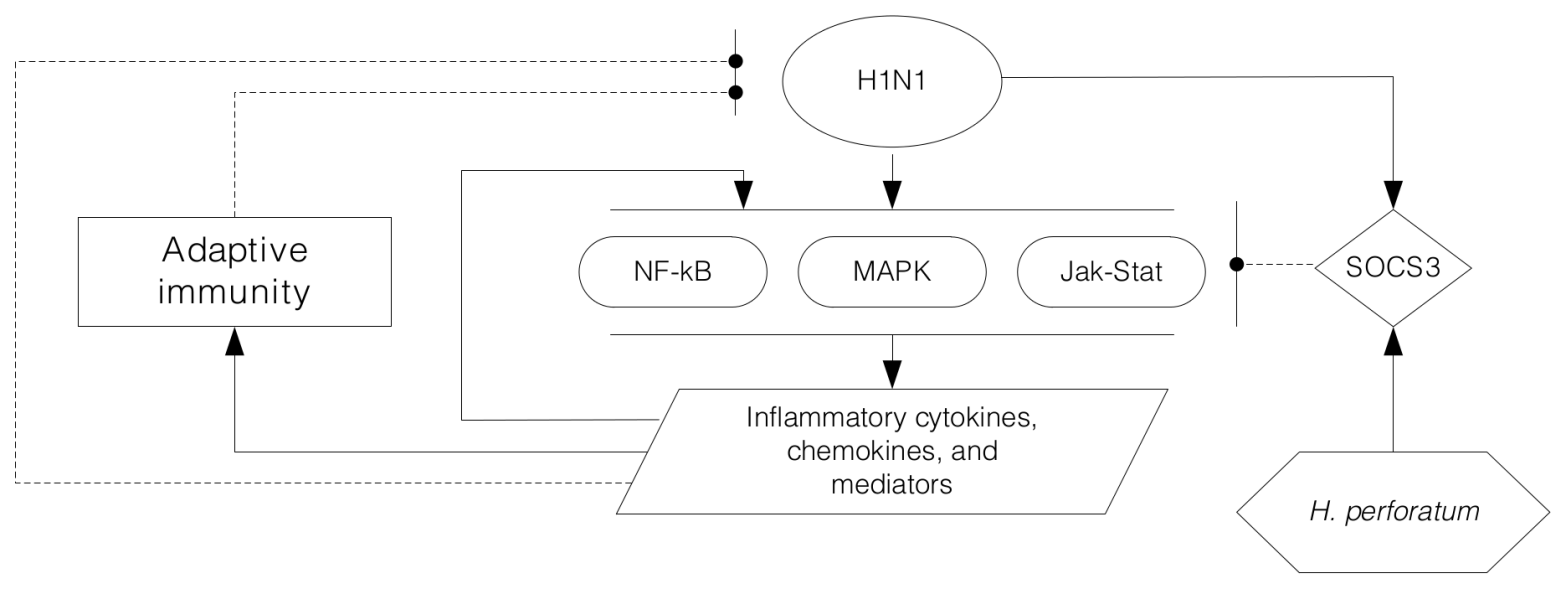
Schematic diagram for the proposed underlying mechanism. SOCS3 was a negative regulator that undermines innate immunity against influenza virus infection. *H. perforatum* extract elevated SOCS3, and thus impaired the required anti-viral immune defense system, resulting in uncontrolled infection and sustained inflammation.

In conclusion, our study revealed an immune-regulatory impact of *H* *. perforatum* extract, which appeared to impair immune defense rather than inhibit inflammation during influenza virus infection. The lower survival rate observed among mice fed with *H* *. perforatum* extract confirmed the potential contraindication of using *H* *. perforatum* during severe influenza illness. The elevated SOCS3 associated with the extract deserves further investigation, as it could be facilitating viral invasion by interfering with innate immune regulation as depicted in Figure **12**.

## Supporting Information

Figure S1 **Cell viability with virus challenge and *H* *. perforatum* extract treatment.** Cell viability was measured by MTS reagent at the time of harvesting supernatant. Results are shown as percentage of not challenged vehicle control (Mean ± SEM, N=3).
(TIF)

Figure S2 **Mouse serum cytokine levels in the 6 day low viral titer inoculation study.** Mice were inoculated with H1N1 virus (10^5.0^ EID50) on day 0 and gavaged with 5% ethanol vehicle control from day -1 to day 5 (N=12), or inoculated with H1N1 virus on day 0 and gavaged with 110 mg/kg *H. perforatum* extract (N=12), or not inoculated by virus and gavaged with 5% ethanol (N=6). Serum IL-6 (**A**) and IL-1β (**B**) were measured using ELISA and shown as Mean ± SEM. Values without same label are different from each other statistically (p<0.05).
(TIF)

## References

[1] Dushoff J, Plotkin JB, Viboud C, Earn DJ, Simonsen L (2006) Mortality due to influenza in the United States--an annualized regression approach using multiple-cause mortality data. Am J Epidemiol 163: 181-187. PubMed: 16319291. 16319291 10.1093/aje/kwj024

[2] Taubenberger JK, Morens DM (2008) The pathology of influenza virus infections. Annu Rev Pathol 3: 499-522. doi:10.1146/annurev.pathmechdis.3.121806.154316. PubMed: 18039138. 18039138 10.1146/annurev.pathmechdis.3.121806.154316 PMC2504709

[3] Mordstein M, Kochs G, Dumoutier L, Renauld JC, Paludan SR et al. (2008) Interferon-lambda contributes to innate immunity of mice against influenza A virus but not against hepatotropic viruses. PLOS Pathog 4: e1000151. 18787692 10.1371/journal.ppat.1000151 PMC2522277

[4] Peiris JS, Cheung CY, Leung CY, Nicholls JM (2009) Innate immune responses to influenza A H5N1: friend or foe? Trends Immunol 30: 574-584. doi:10.1016/j.it.2009.09.004. PubMed: 19864182. 19864182 10.1016/j.it.2009.09.004 PMC5068224

[5] Sanders CJ, Doherty PC, Thomas PG (2010) Respiratory epithelial cells in innate immunity to influenza virus infection. Cell Tissue Res, 343: 13–21. PubMed: 20848130. 20848130 10.1007/s00441-010-1043-z

[6] McGill J, Heusel JW, Legge KL (2009) Innate immune control and regulation of influenza virus infections. J Leukoc Biol 86: 803-812. doi:10.1189/jlb.0509368. PubMed: 19643736. 19643736 10.1189/jlb.0509368 PMC2752015

[7] Kobasa D, Jones SM, Shinya K, Kash JC, Copps J et al. (2007) Aberrant innate immune response in lethal infection of macaques with the 1918 influenza virus. Nature 445: 319-323. doi:10.1038/nature05495. PubMed: 17230189. 17230189 10.1038/nature05495

[8] Tscherne DM, García-Sastre A (2011) Virulence determinants of pandemic influenza viruses. J Clin Invest 121: 6-13. doi:10.1172/JCI44947. PubMed: 21206092. 21206092 10.1172/JCI44947 PMC3007163

[9] Di Carlo G, Borrelli F, Ernst E, Izzo AA (2001) St John’s wort: Prozac from the plant kingdom. Trends Pharmacol Sci 22: 292-297. doi:10.1016/S0165-6147(00)01716-8. PubMed: 11395157. 11395157 10.1016/s0165-6147(00)01716-8

[10] Paterniti I, Briguglio E, Mazzon E, Galuppo M, Oteri G et al. (2010) Effects of *Hypericum* *Perforatum*, in a rodent model of periodontitis. BMC Complement Altern Med 10: 73. doi:10.1186/1472-6882-10-73. PubMed: 21092263. 21092263 10.1186/1472-6882-10-73 PMC3000377

[11] Birt DF, Widrlechner MP, Hammer KD, Hillwig ML, Wei J et al. (2009) *Hypericum* in infection: Identification of anti-viral and anti-inflammatory constituents. Pharm Biol 47: 774-782. doi:10.1080/13880200902988645. PubMed: 19907671. 19907671 10.1080/13880200902988645 PMC2774925

[12] Berlato C, Cassatella MA, Kinjyo I, Gatto L, Yoshimura A et al. (2002) Involvement of suppressor of cytokine signaling-3 as a mediator of the inhibitory effects of IL-10 on lipopolysaccharide-induced macrophage activation. J Immunol 168: 6404-6411. PubMed: 12055259. 12055259 10.4049/jimmunol.168.12.6404

[13] Baetz A, Frey M, Heeg K, Dalpke AH (2004) Suppressor of cytokine signaling (SOCS) proteins indirectly regulate toll-like receptor signaling in innate immune cells. J Biol Chem 279: 54708-54715. doi:10.1074/jbc.M410992200. PubMed: 15491991. 15491991 10.1074/jbc.M410992200

[14] Rawlings JS, Rosler KM, Harrison DA (2004) The JAK/STAT signaling pathway. J Cell Sci 117: 1281-1283. doi:10.1242/jcs.00963. PubMed: 15020666. 15020666 10.1242/jcs.00963

[15] Qasimi P, Ming-Lum A, Ghanipour A, Ong CJ, Cox ME et al. (2006) Divergent mechanisms utilized by SOCS3 to mediate interleukin-10 inhibition of tumor necrosis factor alpha and nitric oxide production by macrophages. J Biol Chem 281: 6316-6324. doi:10.1074/jbc.M508608200. PubMed: 16352613. 16352613 10.1074/jbc.M508608200

[16] Yasukawa H, Ohishi M, Mori H, Murakami M, Chinen T et al. (2003) IL-6 induces an anti-inflammatory response in the absence of SOCS3 in macrophages. Nat Immunol 4: 551-556. doi:10.1038/ni938. PubMed: 12754507. 12754507 10.1038/ni938

[17] Huang N, Rizshsky L, Hauck CC, Nikolau BJ, Murphy PA et al. (2012) The inhibition of lipopolysaccharide-induced macrophage inflammation by 4 compounds in *Hypericum* *perforatum* extract is partially dependent on the activation of SOCS3. Phytochemistry 76: 106-116. doi:10.1016/j.phytochem.2011.12.001. PubMed: 22245632. 22245632 10.1016/j.phytochem.2011.12.001 PMC3294117

[18] Pothlichet J, Chignard M, Si-Tahar M (2008) Cutting edge: innate immune response triggered by influenza A virus is negatively regulated by SOCS1 and SOCS3 through a RIG-I/IFNAR1-dependent pathway. J Immunol 180: 2034-2038. PubMed: 18250407. 18250407 10.4049/jimmunol.180.4.2034

[19] Hammer KD, Yum MY, Dixon PM, Birt DF (2010) Identification of JAK-STAT pathways as important for the anti-inflammatory activity of a *Hypericum* *perforatum* fraction and bioactive constituents in RAW 264.7 mouse macrophages. Phytochemistry 71: 716-725. 20303133 10.1016/j.phytochem.2010.02.006 PMC2858624

[20] Davis JM, Murphy EA, McClellan JL, Carmichael MD, Gangemi JD (2008) Quercetin reduces susceptibility to influenza infection following stressful exercise. Am J Physiol Regul Integr Comp Physiol 295: R505-R509. doi:10.1152/ajpregu.90319.2008. PubMed: 18579649. 18579649 10.1152/ajpregu.90319.2008

[21] Sim YJ, Yu S, Yoon KJ, Loiacono CM, Kohut ML (2009) Chronic exercise reduces illness severity, decreases viral load, and results in greater anti-inflammatory effects than acute exercise during influenza infection. J Infect Dis 200: 1434-1442. doi:10.1086/606014. PubMed: 19811098. 19811098 10.1086/606014 PMC2812897

[22] Huang N, Hauck C, Yum MY, Rizshsky L, Widrlechner MP et al. (2009) Rosmarinic acid in Prunella vulgaris ethanol extract inhibits lipopolysaccharide-induced prostaglandin E2 and nitric oxide in RAW 264.7 mouse macrophages. J Agric Food Chem 57: 10579-10589. 19919113 10.1021/jf9023728 PMC2795400

[23] Viksman MY, Liu MC, Bickel CA, Schleimer RP, Bochner BS (1997) Phenotypic analysis of alveolar macrophages and monocytes in allergic airway inflammation. I. Evidence for activation of alveolar macrophages, but not peripheral blood monocytes, in subjects with allergic rhinitis and asthma. Am J Respir Crit Care Med 155: 858-863. doi:10.1164/ajrccm.155.3.9117017. PubMed: 9117017. 9117017 10.1164/ajrccm.155.3.9117017

[24] Davidson BA, Stewart CC, Russo TA, Chess PR, Knight PR 3rd (2005) Discrimination of resident and infiltrated alveolar macrophages by flow cytometry in influenza A virus-infected mice. Exp Lung Res 31: 323-339. doi:10.1080/01902140590918524. PubMed: 15962712. 15962712 10.1080/01902140590918524

[25] Liu Z, Yang ZQ, Xiao H (2010) Antiviral activity of the effective monomers from Folium Isatidis against influenza virus in vivo. Virol Sin 25: 445-451. doi:10.1007/s12250-010-3142-0. PubMed: 21221924. 21221924 10.1007/s12250-010-3142-0 PMC8227894

[26] Maus UA, Herold S, von Wulffen W, Steinmueller M, Pleschka S et al. (2006) Alveolar epithelial cells direct monocyte transepithelial migration upon influenza virus infection: Impact of chemokines and adhesion molecules. J Immunol 177: 1817-1824. PubMed: 16849492. 16849492 10.4049/jimmunol.177.3.1817

[27] Pamer EG (2009) Tipping the balance in favor of protective immunity during influenza virus infection. Proc Natl Acad Sci U S A 106: 4961-4962. doi:10.1073/pnas.0901574106. PubMed: 19321752. 19321752 10.1073/pnas.0901574106 PMC2664038

[28] Huang N, Rizshsky L, Hauck C, Nikolau BJ, Murphy PA et al. (2011) Identification of anti-inflammatory constituents in *Hypericum* *perforatum* and *Hypericum* *gentianoides* extracts using RAW 264.7 mouse macrophages. Phytochemistry 72: 2015-2023. 21855951 10.1016/j.phytochem.2011.07.016 PMC3197739

[29] Calapai G, Crupi A, Firenzuoli F, Inferrera G, Ciliberto G et al. (2001) Interleukin-6 involvement in antidepressant action of *Hypericum* *perforatum* . Pharmacopsychiatry 34 Suppl 1: S8-10. doi:10.1055/s-2001-15507. PubMed: 11518082. 11518082 10.1055/s-2001-15507

[30] Grundmann O, Lv Y, Kelber O, Butterweck V (2010) Mechanism of St. John's wort extract (STW3-VI) during chronic restraint stress is mediated by the interrelationship of the immune, oxidative defense, and neuroendocrine system. Neuropharmacology 58: 767-773. doi:10.1016/j.neuropharm.2009.12.014. PubMed: 20036263. 20036263 10.1016/j.neuropharm.2009.12.014

[31] Pauli EK, Schmolke M, Wolff T, Viemann D, Roth J et al. (2008) Influenza A virus inhibits type I IFN signaling via NF-kappaB-dependent induction of SOCS-3 expression. PLOS Pathog 4: e1000196 PubMed: 18989459. 18989459 10.1371/journal.ppat.1000196 PMC2572141

[32] Gao H, Hoesel LM, Guo RF, Rancilio NJ, Sarma JV et al. (2006) Adenoviral-mediated overexpression of SOCS3 enhances IgG immune complex-induced acute lung injury. J Immunol 177: 612-620. PubMed: 16785559. 16785559 10.4049/jimmunol.177.1.612

[33] Hartshorn KL, Karnad AB, Tauber AI (1990) Influenza A virus and the neutrophil: a model of natural immunity. J Leukoc Biol 47: 176-186. PubMed: 2406357. 2406357 10.1002/jlb.47.2.176

[34] Herold S, Steinmueller M, von Wulffen W, Cakarova L, Pinto R et al. (2008) Lung epithelial apoptosis in influenza virus pneumonia: the role of macrophage-expressed TNF-related apoptosis-inducing ligand. J Exp Med 205: 3065-3077. doi:10.1084/jem.20080201. PubMed: 19064696. 19064696 10.1084/jem.20080201 PMC2605231

[35] Wareing MD, Lyon AB, Lu B, Gerard C, Sarawar SR (2004) Chemokine expression during the development and resolution of a pulmonary leukocyte response to influenza A virus infection in mice. J Leukoc Biol 76: 886-895. doi:10.1189/jlb.1203644. PubMed: 15240757. 15240757 10.1189/jlb.1203644

[36] Tate MD, Deng YM, Jones JE, Anderson GP, Brooks AG et al. (2009) Neutrophils ameliorate lung injury and the development of severe disease during influenza infection. J Immunol 183: 7441-7450. doi:10.4049/jimmunol.0902497. PubMed: 19917678. 19917678 10.4049/jimmunol.0902497

